# Tetraspanin CD82 Inhibits Protrusion and Retraction in Cell Movement by Attenuating the Plasma Membrane-Dependent Actin Organization

**DOI:** 10.1371/journal.pone.0051797

**Published:** 2012-12-14

**Authors:** Wei M. Liu, Feng Zhang, Simon Moshiach, Bin Zhou, Chao Huang, Kamalakkannan Srinivasan, Seema Khurana, Yi Zheng, Jill M. Lahti, Xin A. Zhang

**Affiliations:** 1 Vascular Biology and Cancer Centers and Departments of Medicine and Molecular Science, University of Tennessee Health Science Center, Memphis, Tennessee, United States of America; 2 Department of Genetics and Tumor Cell Biology, St. Jude Children’s Research Hospital, Memphis, Tennessee, United States of America; 3 Department of Physiology, University of Tennessee Health Science Center, Memphis, Tennessee, United States of America; 4 Division of Experimental Hematology, Cincinnati Children's Hospital, Cincinnati, Ohio, United States of America; Institute of Molecular and Cell Biology, Singapore

## Abstract

To determine how tetraspanin KAI1/CD82, a tumor metastasis suppressor, inhibits cell migration, we assessed which cellular events critical for motility are altered by KAI1/CD82 and how KAI1/CD82 regulates these events. We found that KAI1/CD82-expressing cells typically exhibited elongated cellular tails and diminished lamellipodia. Live imaging demonstrated that the polarized protrusion and retraction of the plasma membrane became deficient upon KAI1/CD82 expression. The deficiency in developing these motility-related cellular events was caused by poor formations of actin cortical network and stress fiber and by aberrant dynamics in actin organization. Rac1 activity was reduced by KAI1/CD82, consistent with the diminution of lamellipodia and actin cortical network; while the growth factor-stimulated RhoA activity was blocked by KAI1/CD82, consistent with the loss of stress fiber and attenuation in cellular retraction. Upon KAI1/CD82 expression, Rac effector cofilin was not enriched at the cell periphery to facilitate lamellipodia formation while Rho kinase exhibited a significantly lower activity leading to less retraction. Phosphatidylinositol 4, 5-biphosphate, which initiates actin polymerization from the plasma membrane, became less detectable at the cell periphery in KAI1/CD82-expressing cells. Moreover, KAI1/CD82-induced phenotypes likely resulted from the suppression of multiple signaling pathways such as integrin and growth factor signaling. In summary, at the cellular level KAI1/CD82 inhibited polarized protrusion and retraction events by disrupting actin reorganization; at the molecular level, KAI1/CD82 deregulated Rac1, RhoA, and their effectors cofilin and Rho kinase by perturbing the plasma membrane lipids.

## Introduction

Regulating cell motility is a common feature of many tetraspanins [1]–[4]. Although it remains largely unclear how tetraspanins modulate cell motility, possible mechanisms have started to emerge from recent research [5]–[7]. Lines of evidence suggest that tetraspanins could regulate the functional status of cell adhesion molecules and growth factor receptors (or membrane-bound growth factor) that they physically and/or functionally associate with and then alter cellular behaviors through these partners [1]–[7]. For example, KAI1/CD82 attenuates epidermal growth factor (EGF) signaling and integrin function by accelerating endocytosis of its associated EGF receptor and integrin, respectively [8], [9]. In parallel, experimental data also support the notion that tetraspanins *per se* solicit outside-in signals to modulate cellular functions [1]–[4]. Again, taking KAI1/CD82 as an example, immuno-crosslinking of cell surface KAI1/CD82 demonstrates that KAI1/CD82 functions as a costimulatory molecule during T cell activation [10]–[15], indicating that KAI1/CD82 plays a direct role in signal initiation and/or transduction.

Regardless of which of the two possible mechanisms plays a more predominant role, tetraspanins and/or their associated molecules must act on cytoskeleton to alter motility-related cellular events and ultimately affect cell motility. For example, clustering the cell surface KAI1/CD82 proteins by using immobilized KAI1/CD82 monoclonal antibody (mAb) induces profound dendritic cellular processes in T cells, accompanied by the rearrangement of actin cytoskeleton and the connection of KAI1/CD82 to actin cytoskeleton, in a protein kinase A activity-dependent, but Src kinase activity-independent, manner [12], [15]. Further studies have indicated that Rho small GTPases are required for KAI1/CD82-induced dendritic processes in T cells [13].

Cell migration requires the polarized formation and extension of cellular protrusions, the transmembrane connection of cytoskeleton to extracellular matrix (ECM) to generate traction force to propel the cell body forward, and the retraction of the rear cellular portion [16]. Thus, by nature, cell migration is a process of global reorganization of cytoskeleton. For example, actin polymerization drives the formation and extension of the protrusions such as lamellipodia at the leading edge [17]–[20], while the asymmetric distribution and enzymatic engagement of myosin and actin produce the force for cellular contractility and lead to the retraction of the trailing edge [21]–[23]. Rho small GTPases are clearly pivotal in all of these cytoskeletal rearrangement processes [16]. For example, Rac is primarily responsible for generating a protrusive force through the localized actin polymerization, while Rho is responsible for the contraction of the cell body and the retraction of the rear end [24]. As downstream effectors of Rho GTPases [16], cofilin severs actin filament to generate barbed ends and thus facilitates the actin treadmilling [16], [25] while Arp2/3 complex nucleates new actin filaments from the sides of preexisting filaments [16], [26]. The severing activity of cofilin and branching activity of Arp2/3 function coordinately to promote the formation of a branched actin network or cortical actin meshwork at the leading edge and generate propulsive force for migrating cells [27]. The activation of Rho kinase, an effector of RhoA, leads to increased myosin phosphorylation and actomyosin contractility and therefore is required for the retraction process during cell movement [16].

Tetraspanin KAI1/CD82 is an inhibitor of cell movement [28]–[38]. Recent studies have revealed that, in solid tumor cells, KAI1/CD82 attenuates the signaling derived from integrin [39], epidermal growth factor receptor (EGFR) [40], and c-Met [41], [42] and that the FAK-Src-p130^CAS^-Crk pathway is a major downstream signaling pathway affected by KAI1/CD82 [30], [42]. At the cellular level, besides the aforementioned induction of dendritic cellular processes via immobilized KAI1/CD82 mAbs, earlier studies from us and elsewhere showed that KAI1/CD82 inhibits lamellipodia formation [9], [43]. The cellular and molecular events critical for the function of KAI1/CD82 and the signaling altered by KAI1/CD82, however, have not been elucidated in detail. The goal of this study was to determine the subcellular event and cytoskeletal event crucial for the motility-inhibitory activity of KAI1/CD82. In this study, we found that KAI1/CD82 inhibits both protrusion and retraction events, which result from the deficient development of actin cortical meshwork and stress fibers. Not surprisingly, actin polymerization becomes attenuated upon KAI1/CD82 overexpression because of the deregulation of Rho small GTPase activities and aberrant functions of Rho GTPase effectors.

## Materials and Methods

### Reagents

The mAbs used in this study were human integrin α3 mAb X8 [44], human integrin α5 mAb PUJ-2 [45], human integrin β1 mAb A1A5 and TS2/16 [46], human CD81 mAb M38 [47], human KAI1/CD82 mAbs M104 [47], 4F9 [14], [48], and TS82b (Tepnel, Stamford, CT), phosphotidylinositol 4, 5-biphosphate (PIP_2_) mAb (Assay Designs Inc., Ann Arbor, MI), and β-tubulin mAb (Sigma, St. Louis, MO). A mouse monoclonal IgG2 was used as a negative control antibody (Sigma). The polyclonal Ab (pAb) for cofilin was purchased from Cytoskeleton (Denver, CO), for phosphorylated cofilin from Dr. J. Bamburg from Colorado State University or Cell Signalling (Danvers, MA), and for the p34 of Arp2/3 complex from Upstate Biotechnology (Lake Placid, NY). The mAbs and/or pAbs for RhoA, Cdc42, Rac1, vinculin, and paxillin were purchased from Santa Cruz Biotechnology (Santa Cruz, CA) or BD Bioscience (San Jose, CA). The secondary antibodies were horseradish peroxidase-conjugated goat anti-mouse or -rabbit IgG antibody (Sigma) and rhodamine-conjugated goat-anti-mouse IgG antibody (Biosource International, Camarillo, CA).

Extracellular matrix (ECM) proteins used in this study were human plasma fibronectin (FN) (Invitrogen, San Diego, CA), mouse laminin (LN)-1 (Invitrogen, San Diego, CA), and rat LN-5 (Desmos, Inc., San Diego, CA).

Growth factors or chemokines used in this study were epidermal growth factor (EGF) (Upstate Biotechnology, Lake Placid, NY), platelet-derived growth factor (PDGF) (Upstate Biotechnology), hepatocyte growth factor (HGF) (Sigma), and stromal cell-derived factor 1 (SDF-1) (R&D, Minneapolis, MN).

Alexa 488- or Alexa 594-conjugated phalloidin was obtained from Invitrogen.

### Transfectants

Prostate cancer cell lines Du145 and PC3 were obtained from ATCC (Manassas, VA) and cultured in DMEM supplemented with 10% FBS, 100 units/ml penicillin, and 100 µg/ml streptomycin. The full-length KAI1/CD82 cDNA was constructed in a eukaryotic expression vector pCDNA3.1 (Invitrogen). Du145 or PC3 cells were transfected with the plasmid DNA by using either Superfectin (Qiagen, Valencia, CA) or Lipofectamine 2000 (Invitrogen), respectively, and selected with 1 mg/ml geneticin (Invitrogen). The geneticin-resistant clones were pooled, and the KAI1/CD82-positive cells were collected by flow cytometric cell sorting [30].

For KAI1/CD82 silencing, HT29 human colon cancer cells (ATCC) were transiently transfected with KAI1/CD82-specific small interfering RNA (siRNA) duplex pool or control siRNA duplex (Dharmacon, Lafayette, CO) by using Lipofectamine RNAiMAX (Invitrogen). The transfected cells were sparsely seeded on glass coverslips and used for experiments 48 h after transfection.

In some experiments, plasmid DNA of pEGFP, pEGFP-actin (kindly provided by Dr. A. Weaver of Vanderbilt University), pEGFP-WASP (kindly provided by Dr. Alissa Weaver of Vanderbilt), and pEGFP-PLCδ PH domain (kindly provided by Dr. John Cox of the University of Tennessee) were transiently transfected into Du145-KAI1/CD82 and -Mock cells by using Lipofectamine 2000 and following the manufacturer’s protocol.

### Time-lapse Videomicroscopy

The time-lapse videomicroscopy experiments were performed basically as previously described [49], [50]. The Du145- or PC3-transfectant cells were plated on ECM protein (FN or LN1)-coated glass bottom dishes (MatTek Corp., Ashland, MA) in DMEM medium from 3 to 6 h before the time-lapse imaging experiment. In the imaging experiment, each transfectant cell was observed from 1 to 6 h on an Axiovert 135 TV microscope (Carl Zeiss, Thornwood, NY) with DIC optics with either an oil-immersion 40X Plan Fluor or an oil-immersion 63X Plan Apo objective. The microscope was equipped with a heated stage, and the temperature was kept at 37°C. CO_2_ was maintained by covering the medium with mineral oil. Images were taken with a Hamamatsu cooled CCD camera run by Metamorph (Universal Imaging, Pittsburgh, PA) in 1-, 2-, 5-, or 10-min intervals during the observation periods. The cellular motile behaviors and the translocation of cells were assessed in movies made from the saved images. Cellular motile behaviors were analyzed under different conditions such as serum-free DMEM, DMEM containing EGF, DMEM containing KAI1/CD82 mAb, etc. The images were analyzed by Nikon EZ-C1 FreeViewer software.

For the intracellular actin polymerization study, GFP and GFP-actin constructs were transfected 48 h prior to experiments, then plated on FN-coated glass bottom plates (MatTek Corp., Ashland, MA). Images were taken with a C1Si confocal system mounted on an Eclipse TE2000-E microscope (Nikon, Melville, NY), using a Plan Fluor oil-immersion 40X objective (N.A., 1.3) or an oil-immersion Plan Apo 60× objective (N.A., 1.45). The system was equipped with an environmental control chamber (InVivo Scientific, St Louis, MO) to keep the environment at 37°C and 5% CO_2_. DIC and fluorescent images were recorded every 1–2 min, and polymerization of GFP-actin was assessed in movies made from the saved images.

### Fluorescent and Confocal Microscopy

The immunofluorescence staining was carried out as previously described [51], [52]. In brief, glass coverslips were coated with either FN (50 μg/ml) in 10 mM NaHCO_3_ or LN 1 (50 μg/ml) in phosphate buffer saline (PBS) at 4°C overnight and then blocked with 0.1% heat-inactivated bovine serum albumin (BSA) at 37°C 45 min. Du145 transfectants were harvested in PBS containing 2 mM EDTA, washed once, and plated on the ECM-coated coverslips in serum-free or serum DMEM from 4 to 6 h at 37°C. Then the cells were fixed with 3% paraformaldehyde (Sigma) and permeated with 0.1% Brij99 (Sigma). Nonspecific binding sites were blocked with 20% goat serum in PBS for 1 h at room temperature (RT). The cells were incubated sequentially with primary mAbs (∼1 μg/ml) and with Rhodamine-conjugated secondary antibody. For F-actin staining, cells were incubated with Texas Red-conjugated phalloidin. Each incubation lasted 30 min at RT in 20% goat serum/PBS and followed by four washes with PBS. Finally, the coverslips were mounted on glass slides in FluroSave reagent (Calbiochem-Novabiochem, San Diego, CA), analyzed using an Axiophot fluorescent microscope (Carl Zeiss) or a Bio-Rad 1024 confocal microscope (Bio-Rad, Hercules, CA), and photographed with an Optronics digital camera (Southern Micro Instrument, Marietta, GA) at 40X or 63× magnification.

### Rho GPTase Activity Assays

The cellular Rho GTPase activities were measured by an effector domain pull-down assay as previously described [53]. Du145 transfectant cells were washed with PBS buffer once and lysed in RIPA buffer (1% NP40, 0.2% SDS, 150 mM NaCl, 25 mM HEPES, 2 mM phenylmethylsulfonyl-fluoride, 20 µg/ml leupeptin, 20 µg/ml aprotinin, 2 mM sodium vanadate, and 2 mM sodium fluoride) on ice. Cell lysates were clarified by centrifugation at 14,000 *g* at 4°C for 5 min, and equal amounts of lysate were incubated with GST fusion beads at 4°C for 45 min. The beads were washed three times with ice-cold RIPA buffer. To assay the activity of Cdc42, cell lysate was affinity-precipitated with the beads absorbed with the Wiskott-Aldrich syndrome protein (WASP) Cdc42 binding domain-GST fusion that binds only to the active GTP-bound form of Cdc42. With the same principle, Rac1 or RhoA activity was pulled down with p21-activated kinase (PAK) Rac1 binding domain-GST fusion or Rhotekin RhoA binding domain-GST fusion beads, respectively. The total cell lysates and the affinity-precipitated products were run on an SDS-PAGE gel, transferred to nitrocellulose membrane, and then immunoblotted for Cdc42, Rac1, or RhoA, respectively. The blots were detected with chemiluminesence reagent (Perkin-Elmer, Boston, MA). The intensities of the bands from four separate experiments were measured by NIH Scion Image program [54].

### Rho Kinase (ROCK) Activity Assay

The ROCK activities in the transfectant cells were measured by using MBL Rho Kinase Assay Kit (MBL International, Woburn, MA). The phosphorylation of Thr^696^ residue in the human myosin phosphatase myosin-binding subunit by the ROCKs from cell lysates was detected in ELISA with the horseradish peroxidase-conjugated AF20 mAb that is specifically against the Thr^696^-phosphorylated myosin-binding subunit peptide. The manufacturer’s protocol was followed for the assay.

### Actin Polymerization Analyses

As described above, intracellular GFP-actin polymerization was analyzed using live fluorescent imaging under a time-lapse confocal microscope. In addition, intracellular F-actin level was measured by using flow cytometry [55]. Briefly, Du145 transfectant cells at the confluent culture stage were detached with trypsin and washed once with sterile PBS. The cells were permeabilized with 0.01% Triton X-100 in PBS at 4°C for 1 min and then washed three times with PBS. The permeabilized cells were fixed with 3.7% formaldehyde at RT for 5 min and subsequently washed three times with PBS. For F-actin staining, the cells were incubated with 66 nM Alexa 488-conjugated phalloidin in PBS containing 1% BSA at 37°C for 1 h. The stained cells were washed three times with PBS and analyzed in a BD LSR II flow cytometer. The flow cytometry data were analyzed using FlowJo 7.1 or FCS express V3 software.

### Western Blot

Western blot was performed as previously described [30]. For total cellular proteins, an equivalent number of cells were lysed using RIPA buffer, the protein concentrations of lysates were normalized, and then the lysates were separated by SDS-PAGE. After being transferred electrically, nitrocellulose membranes (Schleicher & Schuell, Keene, NH) were sequentially blotted with primary antibody and horseradish peroxidase-conjugated anti-mouse or -rabbit IgG (Sigma) and then detected with chemiluminesence reagent (PerkinElmer Life Sciences). In some cases, membranes were stripped and reblotted with mAbs or pAbs according to the manufacturer's instruction.

### Image Analysis

For quantitation of F-actin intensity, the cell perimeters were outlined as described above. Then, based on the fluorescent digital images of α-phalloidin staining, the total cellular F-actin intensity, F-actin intensity inside the inner edge of the cortical actin ring, and F-actin intensity of membrane protrusion or outside the inner edge of the cortical actin ring were measured using the “analyze” function in Image J software. Theoretically, total cellular F-actin intensity = F-actin intensity inside the inner edge of the cortical actin ring + F-actin intensity of membrane protrusion. Relative F-actin intensity of membrane protrusion = F-actin intensity of membrane protrusion/total cellular F-actin intensity (Figure S1).

## Results

### The Aberrant Cell Morphology Induced by KAI1/CD82 Results from the Deficiencies in Retraction of Cellular Tails and Formation of Lamellipodia

**Figure 1:**
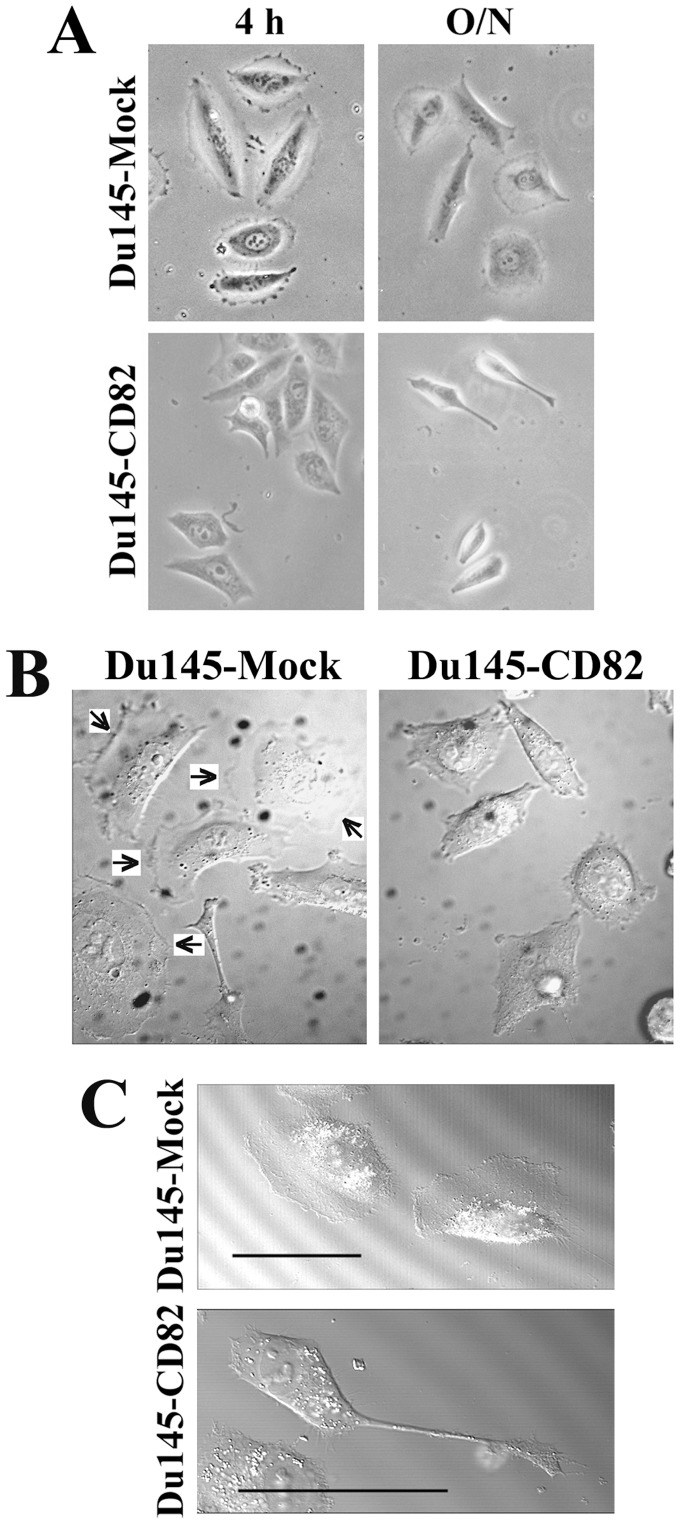
Morphological phenotypes of KAI1/CD82-overexpressing cells. (***A***) KAI1/CD82-expressing cells displayed altered morphology. Du145-Mock and -KAI1/CD82 transfectant cells were plated on FN-coated (10 µg/ml) glass coverslips at 37°C, 5% CO_2_ for 4 h or overnight in serum-free DMEM medium. (***B***) Diminished lamellipodia in KAI1/CD82-overexpressing cells. Du145-Mock and -KAI1/CD82 cells were spread on FN-coated (10 µg/ml) plates in DMEM containing 1% FCS at 37°C overnight. The DIC images of cells were captured on an Axiovert inverted microscope using DIC optics with a 40 x F Fluor oil immersion objective. The arrows indicate lamellipodia. (***C***) KAI1/CD82-overexpressing cells frequently exhibit elongated cellular extensions. Du145-Mock and -KAI1/CD82 cells were spread on FN-coated (10 µg/ml) plates in DMEM containing 10% FCS and HGF (100 ng/ml) at 37°C for 6 h. The DIC images of cells were captured using DIC optics with a 40 x F Fluor oil immersion objective. Scale bar, 50 µm.

Du145 prostate cancer cells barely express any endogenous KAI1/CD82 (Figure S2) [8], [30]. The cellular level of KAI1/CD82 proteins in Du145-CD82 transfectant did not exceed the expression level of endogenous KAI1/CD82 in an immortalized human prostate epithelial cell (PrEC) line (Figure S2). When spread on ECM such as FN, Du145-KAI1/CD82 cells exhibited profound differences in cellular morphology from Du145-Mock cells. KAI1/CD82 overexpression typically results in dumbbell, water-drop, spindle, and irregular polygonal cell shapes (Figure 1A). Such morphological differences exist even when the cells spread in the media that contain fetal calf serum or growth factors. The same alterations in morphology were also observed in the PC3 prostate cancer cells in which KAI1/CD82 was overexpressed (Figure S3). Similar to Du145 cells, PC3 cells either do not express KAI1/CD82 or express a low level of KAI1/CD82 [43].

**Figure 2:**
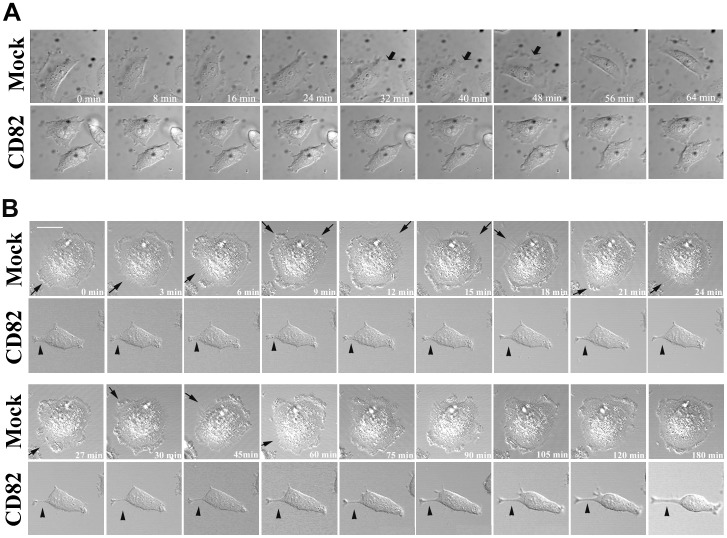
KAI1/CD82 attenuates the formation of lamellipodia and retraction of cellular tails. (***A***) KAI1/CD82 inhibits lamellipodia and protrusion formations. Du145-Mock and -KAI1/CD82 transfectant cells were plated on FN-coated glass coverslips from 3 to 6 h. DIC images were acquired using time-lapse vidoemicroscopy. Arrows indicate lamellipodia. (***B***). KAI1/CD82 inhibits the retraction of the rear tail. Du145-Mock and -KAI1/CD82 cells were placed on FN-coated (10 µg/ml) coverslips from 3 to 6 h and treated with 100 ng/ml HGF from 4 to 6 h. Cell morphology was photographed for 3 h using time-lapse videomicroscopy. Arrows indicate the retraction processes in Du145-Mock cells and arrowheads indicate the rear tail in Du145-KAI1/CD82 cells. The time-lapse intervals are labeled inside images. Scale bar, 20 µm.

In KAI1/CD82-expressing cells with multilateral morphology, cell edges usually became flat or slightly concave, reflecting the lack of lamellipodia formation. As reported earlier by us and others [9], [43], [56], the forced expression of KAI1/CD82 inhibits lamellipodia formation. In Du145-KAI1/CD82 cells, lamellipodia formation was completely lost (Figure 1B); while in PC3-KAI1/CD82 cells, localized lamellipodia were typically limited to the one or two cellular ends, in contrast to widespread and fan-like lamellipodia in PC3-Mock cells (Figure S3B). The diminished lamellipodia could result from either less protrusive or more retraction activities at the cell periphery during cell spreading and migration. To determine which step was affected by KAI1/CD82, we analyzed lamellipodia formation with live imaging. Time-lapse videomicroscopy demonstrated that Du145-Mock cells could rapidly generate broad lamellipodia, while the KAI1/CD82-expressing cells failed to protrude the cell edges to produce lamellipodia (Figure 2A). Because of reduced protrusive activities, some KAI1/CD82-expressing cells displayed more rigid or concave edges.

In dumbbell-, spindle- and water-drop-like cells, the elongated cellular extension is another predominant morphological change upon KAI1/CD82 overexpression. For example, extraordinarily long cellular extensions were observed in KAI1/CD82-expressing cells treated with HGF (Figure 1C). These cellular extensions could also be observed when cells spread or migrated on substrata coated with FN or LN1 (Figures 2B and S3B) in the presence or absence of serum or growth factor. Although the elongated cellular extensions can also be found in Mock cells, the frequency of occurrence was much lower than in KAI1/CD82-expressing cells. For example, approximately 27% of CD82 cells exhibited extremely long extensions on LN 1 while only about 8% of Mock cells showed the same phenotype (Student’s *t*-test, n = 3, p<0.01). The elongated cellular extensions could result from enhanced protrusion or deficient retraction during cell spreading and migration. Time-lapse video microscopy demonstrated that the elongated cellular extensions were caused by a lack of contractile retraction (Figure 2B).

### KAI1/CD82 Inhibits the Formation of Actin Cortical Network and Stress Fibers on ECM

**Figure 3:**
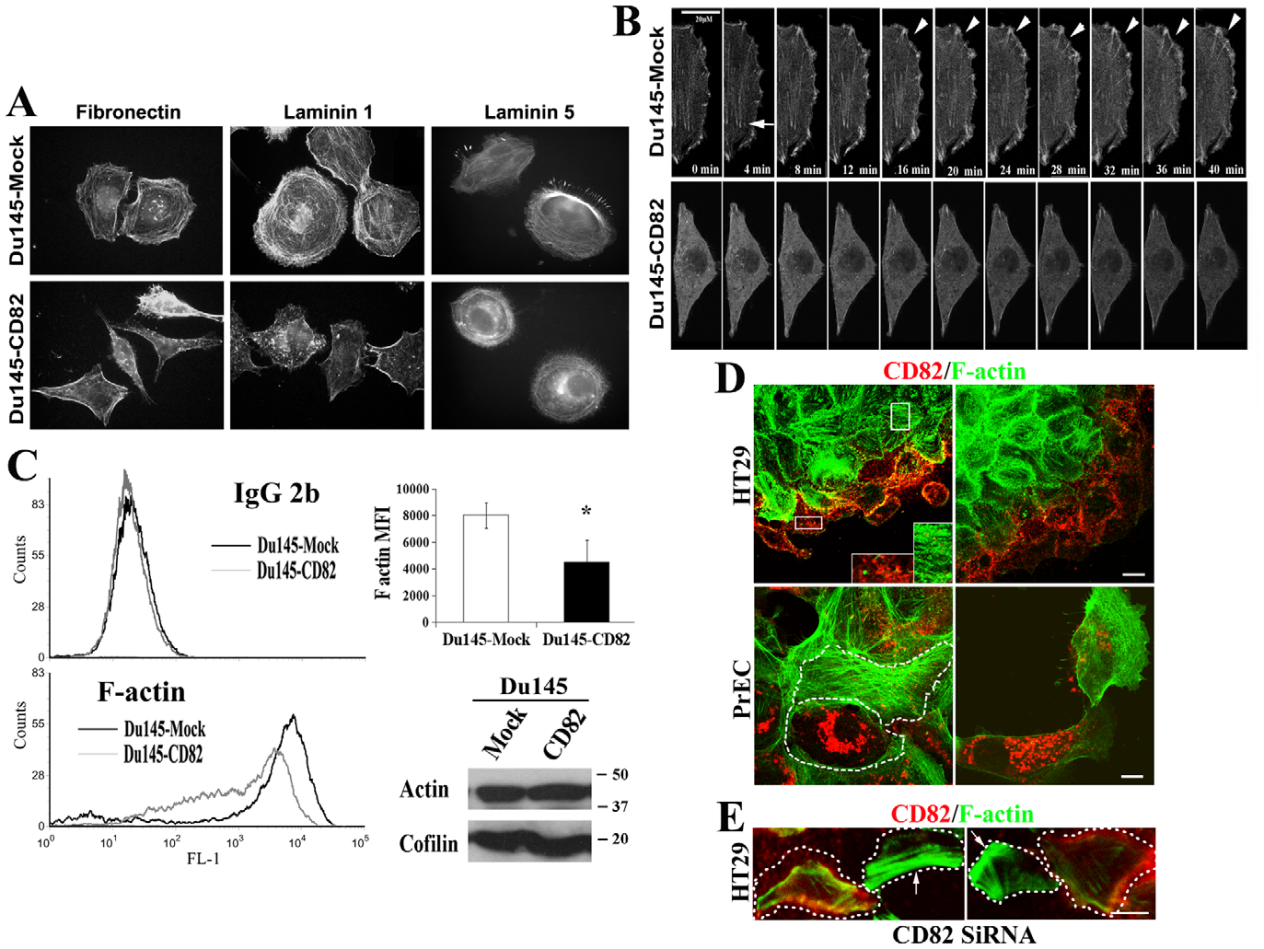
The actin cortical meshwork and stress fiber were disrupted upon KAI1/CD82 expression. (***A*** *)* F-actin distribution. After being spread on FN (50 µg/ml)-, LN1 (50 µg/ml)-, or LN5 (2 µg/ml)-coated coverslips at 37°C, 5% CO_2_ for 6 h, Du145 transfectant cells were fixed, permeabilized, and then stained with TRITC-conjugated α-phalloidin. The fluorescent images were captured under an Axiophot fluorescent microscope equipped with an Optronics digital camera at magnification 63X. (***B*** *)* Live imaging of actin polymerization in Du145-Mock and -KAI1/CD82 cells. The cells that were transiently transfected with pEGFP-actin construct were spread on FN-coated coverslips in complete DMEM and photographed using time-lapse confocal videomicroscopy. Arrowheads and arrows indicate actin polymerization during the development of peripheral meshwork and stress fiber, respectively. *(* ***C*** *)* Less F-actin in Du145-KAI1/CD82 cells. *Left panel*, Du145-Mock and -KAI1/CD82 cells spread in serum-free DMEM were detached, fixed, permeabilized, incubated with Alexa 488-conjugated phalloidin or mouse IgG2b, and then analyzed with flow cytometry. *Right top panel*, quantitation of the mean fluorescence intensity (MFI) of F-actin. Data are expressed as the mean MFI of three independent experiments (*P*<0.05 between Mock and KAI1/CD82 cells). *Right bottom panel*, the cell lysates from the experiment were analyzed by Western blot for total cellular actin proteins. Cofilin blot was used as the protein loading control. *(* ***D*** *)* The presence of KAI1/CD82 proteins is inversely correlated with actin cytoskeletal assembly. HT29 colon cancer cells and prostate epithelial cells were fixed, permeabilized, and incubated with Alexa 488-conjugated phalloidin and Alexa 594-conjugated KAI1/CD82 mAb. The images were acquired with confocal microscopy. For HT29 cells, the photographs were taken from the basal sections of the cells. Shown in insets is the magnified staining from the frame-selected areas. White dashed lines outline the cell boundary. Scale bars: 10 µM. *(* ***E*** *)* KAI1/CD82 silencing leads to stress fiber formation. HT29 cells were transiently transfected with KAI1/CD82-specific siRNA duplexes, seeded sparsely, and stained with Alexa 488-conjugated phalloidin and Alexa 594-conjugated KAI1/CD82 mAb as described above. Scale bar: 10 µM. White dotted lines outline the cell periphery. Arrows indicate the cells exhibit robust stress fiber formation but no KAI1/CD82 staining.

Dynamic actin reorganization is the hallmark of motile cells [57]. For example, the disassembly of extensive stress fibers and formation of cortical meshwork accompany the enhanced cell migration [57]. It was reported that KAI1/CD82 could alter the F-actin rearrangement either by the immobilized KAI1/CD82 mAb [58] or in response to EGF stimulation [9], [43]. But a thorough analysis of F-actin rearrangement is still lacking. In Du145-Mock cells, actin was extensively polymerized into the continuous fibers when the cells were spread on FN-, LN1-, or LN5-coated substrata (Figure 3A). The actin fibers distributed in cell peripheral areas were assembled into cortical actin ring or meshwork that included dorsal stress fiber and transverse arc, while the ones in the cell central areas were the ventral stress fibers (Figure 3A). In contrast, the F-actin in Du145-KAI1/CD82 cell either was stained as patches or focal points or formed discontinuous fibers (Figure 3A), suggesting the aberrancy in the actin organization that is triggered by cell adhesion. In some Du145-KAI1/CD82 cells, F-actin was concentrated at the cell periphery to form dense actin bundles (Figure 3A), which were found much more frequently in KAI1/CD82-expressing cells than in Mock cells, reflecting the impaired development of cortical meshwork. On LN5, although cortical meshwork was formed in KAI1/CD82-expressing cells, it could not become polarized or as well developed as the one in Mock cells (Figure 3A). Less cortical meshwork and stress fibers were also found in PC3-KAI1/CD82 transfectant cells, compared to PC3-Mock cells (Figure S4). PC3-Mock cells displayed the same morphology and actin organization as the untransfected parental PC3 cells do (Figure S4).

### Cell Adhesion-induced Actin Assembly is Inversely Correlated with KAI1/CD82 Expression

To determine the biochemical nature of the aberrant actin organization in KAI1/CD82-expressing cells, we analyzed actin assembly. Consistent with the F-actin staining at steady state under immunoflurescence microscopy, we found that actin assembly was impaired in Du145-KAI1/CD82 cells spread on FN under live imaging within a 40-min time frame by using GFP-actin as a tracer (Figure 3B). In live imaging, the organization of actin into stress fibers (or ventral stress fibers, indicated by arrow in Figure 3B), cortical ring (or transverse arc), and peripheral meshwork (or dorsal stress fibers, indicated by arrow head in Figure 3B) occurred constantly in Mock cells but was significantly attenuated in KAI1/CD82 cells (Figure 3B).

To confirm this observation, we directly measured and compared the intracellular quantity of F-actin by using fluorescent phalloidin in permeabilized Du145 transfectant cells. As shown in Figure 3C, the F-actin level, reflected by the mean fluorescent intensity of intracellular phalloidin staining, was significantly decreased in KAI1/CD82-expressing cells compared with that in Mock cells. The levels of total actin protein remained equivalent between Du145-Mock and -KAI1/CD82 cells (Figure 3C, bottom panel).

To substantiate these observations, we analyzed the relationship between actin cytoskeleton and KAI1/CD82 in the cells expressing endogenous KAI1/CD82 proteins such as HT29 colon cancer cells and PrECs. In HT29 cells, which grow into multicellular clumps in culture, KAI1/CD82 proteins typically became less or not detectable in the subcellular regions where stress fibers were well formed but enriched in the places with fewer or no stress fibers (Figure 3D). Consistently, the PrECs with higher expression of KAI1/CD82 proteins usually exhibited less development of actin cytoskeleton and vice versa (Figure 3D).

We further determined the effect of KAI1/CD82 silencing on actin cytoskeleton development. As indicated by the arrows in Figure 3E, KAI1/CD82 silencing resulted in the formation of more and thicker stress fibers in the HT29 cells without multicellular contacts, while the HT29 cells with KAI1/CD82 expression contained relatively less actin cytoskeleton. Unlike the disparity of KAI1/CD82 staining in the HT29 cells that form multi-cellular contacts (Figure 3D), KAI1/CD82 signals were usually equivalent and even among the HT29 cells that are relatively isolated from each other (data not shown).

Together, the results from the qualitative live imaging and quantitative flow cytometry experiments agreed with each other and demonstrated the impairment in actin assembly in KAI1/CD82 cells.

### The Roles of Cell Adhesion- and Growth Factor-signaling in KAI1/CD82-induced Actin Cytoskeletal Changes

**Figure 4:**
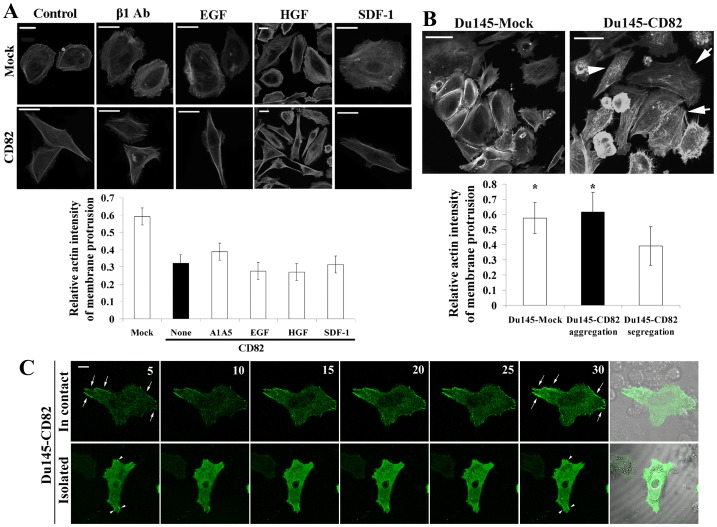
Regulation of KAI1/CD82-induced actin cytoskeletal changes. *(* ***A*** *)* Effects of integrin, growth factor, and chemokine on KAI1/CD82-induced actin cytoskeletal changes. Du145-Mock or -KAI1/CD82 transfectant cells were spread on FN-coated plates in complete DMEM and treated with integrin β1 activating mAb A1A5 (8 µg/ml), EGF (100 ng/ml), HGF (100 ng/ml), or SDF-1 (100 ng/ml) at 37°C from 4 to 6 h. Cells were then fixed, permeabilized, and incubated with Texas Red-conjugated α-phalloidin. Digital images were captured under a fluorescent microscope. Scale bar, 20 µm. Quantitative analysis (*bottom panel*): the cortical F-actin intensity was quantitated as described in Experimental Procedures. Bars denote the average intensity of three individual experiments. In each group, 50∼95 cells were quantified. The differences between Mock and all CD82 groups are statistically significant (*P*<0.05), while the differences between untreated CD82 and each treated CD82 group are not statistically significant (*P*>0.05). (***B***) Effect of the confluent cell culture on KAI1/CD82-induced actin cytoskeletal changes. Actin polymerization was analyzed in the Du145-Mock and -KAI1/CD82 cells cultured in complete DMEM at confluent stage. Scale bars, 100 µm in Du145-Mock cells and 50 µm in Du145-CD82 cells. Arrows indicate the well-developed actin cortical meshwork seen in the KAI1/CD82-expressing cells with cell-cell contacts. Arrowheads indicate that no well-developed cortical network was found in KAI1/CD82-expressing cells without cell-cell contacts. Quantitative analysis (*bottom panel*): the cortical F-actin intensity was quantitated as described in Experimental Procedures. Bars denote the average intensity of 32–40 cells from three individual experiments. The differences between the Mock and KAI1/CD82 cells without cell-cell contacts and between the KAI1/CD82 cells with and without cell-cell contacts are statistically significant (*P*<0.05), while the differences between the groups of Mock and KAI1/CD82 cells containing cell-cell contacts are not statistically significant (*P*>0.05). (***C***) The effect of cell-cell contacts on actin polymerization in Du145-KAI1/CD82 cells. Du145-KAI1/CD82 cells expressing EGFP-actin were photographed using time-lapse confocal and DIC videomicroscopy. For each group, a DIC image is included to display whether the green fluorescent cell is in cell-cell contact. Arrows and arrowheads indicate actin polymerization during the development of peripheral actin fiber. Scale bar: 10 µm.

KAI1/CD82 diminishes the signaling initiated by integrins or growth factors, and these diminutions correlate with KAI1/CD82-induced inhibition of cell movement [59]. To determine which signaling is critical for the effect of KAI1/CD82 on actin, we next investigated whether increased input of the signaling from integrin, growth factor, and chemokine could block, alleviate, or bypass KAI1/CD82-induced changes in actin cytoskeleton. For example, growth factors such as EGF and HGF promote actin cortical meshwork and stress fiber formation or development [60]–[63]. By evaluating the quantity and distribution of F-actin in Du145-KAI1/CD82 cells (as described in Figure S1), we found that the activation of the signaling from β1 integrins, EGFR, c-Met, or CXCR4 either alone (Figure 4A) or in combination (data not shown) could not reverse KAI1/CD82-induced changes such as the loss of actin cortical meshwork or less F-actin in protrusions. Thus, KAI1/CD82 likely affects actin reorganization by inhibiting either multiple signaling pathways or the common signaling step(s) of multiple pathways.

Notably, actin organization in Du145-KAI1/CD82 cells became less abnormal when the cells are cultured in 10% FBS-containing DMEM and at confluence. Under these conditions, actin stress fibers and cortical meshwork were partially or largely restored, respectively, in the KAI1/CD82-expressing cells (Figure 4B). Using live imaging, we analyzed actin assembly in Du145-KAI1/CD82 cells that are either in cell-cell contacts (Figure 4C, top panel) or isolated (Figure 4C, bottom panel). Actin assembly in the cells that form cell-cell contacts, as indicated by the arrows, was markedly more active than that in an isolated cell, as indicated by the arrowheads (Figure 4C). These observations suggest that the signaling resulting from cell-cell adhesion and serum acts against KAI1/CD82 signaling to rescue the actin reorganization defect, probably through either the downstream step(s) of or a pathway parallel to KAI1/CD82 signaling. Alternatively, cell-cell adhesion and serum may reduce the expression of KAI1/CD82 to rescue the defect. By using HT29 cells, which express endogenous KAI1/CD82 and can form multi-cellular spheres in culture, we detected markedly fewer KAI1/CD82 proteins at the center of HT29 spheres, where cells were surrounded by cell-cell contacts (our unpublished data). Temporarily disable cadherin function in confluent HT29 cells with EDTA could not induce the reexpression or reappearance of KAI1/CD82 proteins (data not shown). The physical interactions between CD82 and tetraspanin CD81 remained similar in the Du145-CD82 cells that formed cell-cell contacts (dense) and the ones that were at the isolated growth stage (sparse) (Figure S5). Moreover, HGF was reported to override the motility-inhibitory effect of KAI1/CD82 in YTS1 bladder cancer cells and Du145 cells (41, 42). However, we did not observe a similar effect on cell migration for HGF in Du145-KAI1/CD82 cells (data not shown).

### KAI1/CD82 Regulates Rac1 and RhoA Activities

**Figure 5:**
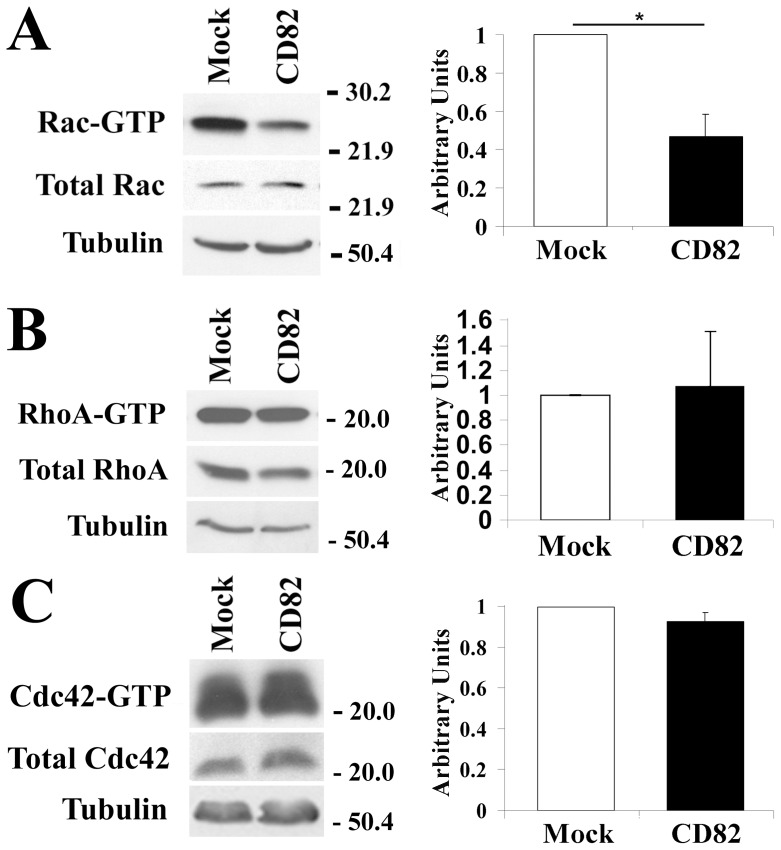
KAI1/CD82 regulates the activities of Rho GTPases. *(* ***A*** *)* KAI1/CD82 inhibits Rac1 activity. Du145-Mock or -KAI1/CD82 transfectant cells were lysed in a lysis buffer containing 1% NP-40 and 0.2% SDS detergents. Cell lysates were subjected to affinity precipitation with GST-PAK1, which binds to only the activated or GTP-bound Rac. The co-precipitated, GTP-bound Rac GTPase was detected by Rac mAb. The intact cell lysates were blotted with Rac mAb to demonstrate equivalent levels of total Rac proteins between Mock and KAI1/CD82 transfectant cells. Blots show the result from a representative experiment; the graph represents the relative density of the Rac band (mean±SD, n = 4), based on densitometric analysis. *: *P*<0.05. (***B***) KAI1/CD82 does not significantly alter RhoA activity. Du145 transfectants were pretreated as described above. GTP-bound RhoA was pulled down by GST-Rhotekin and detected by RhoA mAb. The blot shows the result from a representative experiment; the graph represents the relative density of the RhoA bands (mean±SD, n = 9), based on densitometric analysis. *P*>0.05 between Mock and KAI1/CD82. (***C***) KAI1/CD82 does not significantly alter Cdc42 activity. GTP-bound Cdc42 was pulled down by GST-PAK1 and detected by Cdc42 mAb. The blot shows the results from a representative experiment; the graph represents the relative density of the Cdc42 bands (mean±SD, n = 4), based on densitometric analysis. *P*>0.05 between Mock and KAI1/CD82. In all experiments, tubulin protein levels in cell lysates were detected via Western blot and served as protein loading controls.

Since previous studies found that Rho GTPases are involved in KAI1/CD82-mediated signaling [58] and Rho GTPases are the key regulators of actin cytoskeleton organization [64], [65], we analyzed the Cdc42, Rac, and Rho activities in Du145-Mock and -KAI1/CD82 cells. Using the effector domain pull-down experiments, we found that Rac1 activity was significantly downregulated by KAI1/CD82 (Figure 5). RhoA and Cdc42 activities remained unchanged upon overexpression of KAI1/CD82 (Figure 5). The same results were obtained when the cells were starved from serum overnight. The reduced Rac1 activity in KAI1/CD82-expressing cells could not be upregulated by platelet-derived growth factor, a Rac1 activator (data not shown). The levels of total Rac1, RhoA, and Cdc42 proteins were similar or equivalent between Mock and KAI1/CD82 transfectant cells.

### KAI1/CD82 Blocks the Enrichment of Cofilin at the Cell Periphery and Inhibits ROCK Activity

**Figure 6:**
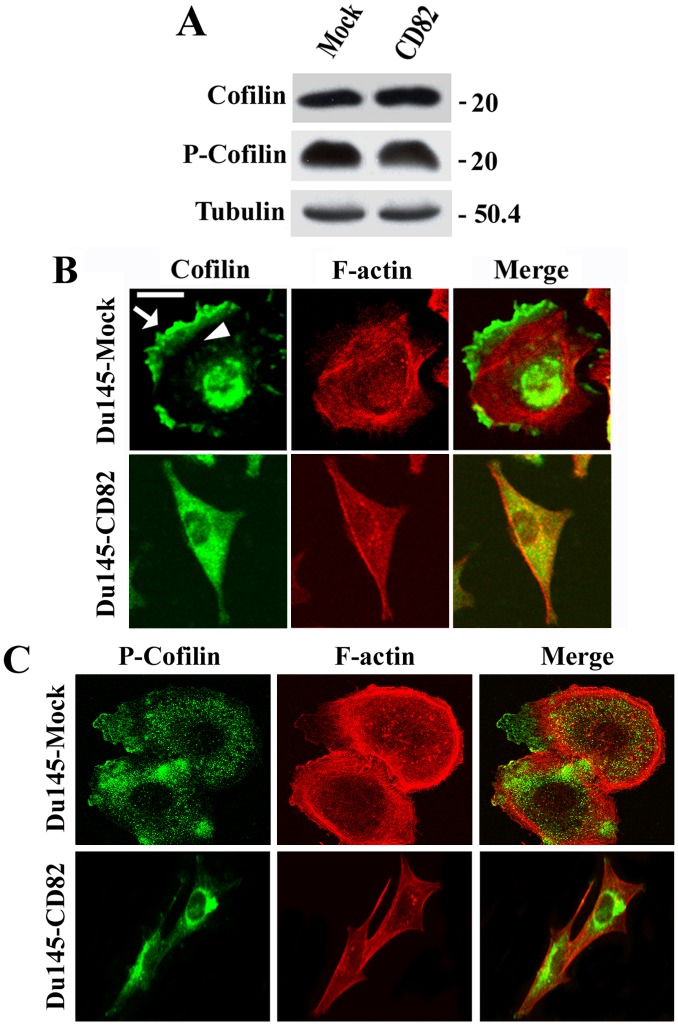
KAI1/CD82 blocks the enrichment of cofilin at the cell periphery. (***A***) The levels of total and phosphorylated cofilin proteins in Du145-Mock and -KAI1/CD82 cells were assessed by Western blot using pAbs against cofilin and phosphorylated cofilin, respectively, as described in Materials and Methods. Tubulin blot is used as a control for protein loading. *(* ***B*** *)* KAI1/CD82 prevents cofilin from being targeted to the cell periphery. Du145 transfectant cells were spread on FN-coated coverslips in complete DMEM from 3 to 6 h. The cells were fixed, permeabilized, and incubated with cofilin pAb and TRITC-conjugated α-phalloidin, followed by the FITC-conjugated second Ab staining. Digital images were captured under a confocal microscope, and each image represents a single XY section. The arrow indicates the translocation of cofilin into lamellipodia, while the arrowhead indicates the relatively transparent zone beneath the actin cortical meshwork and within the cytoplasm. Scale bar, 20 µm. *(* ***C*** *)* Comparison of the subcellular distribution of phosphorylated cofilin between Du145-Mock and -KAI1/CD82 cells. The experiment was performed as described in *(* ***B*** *)* except that the pAb against phosphorylated cofilin was used as the primary Ab. Scale bar, 20 µm.

Cofilin is a downstream effector of both Rac and Rho and promotes actin reorganization [16]. The levels of total, inactive (phosphorylated), and active (unphosphorylated = total - phosphorylated) cofilin proteins remained unchanged in KAI1/CD82-overexpressing cells (Figure 6A). In Mock cells, cofilin was concentrated in membrane protrusions such as lamellipodia (Figure 6B, arrow) and in nuclei (Figure 6B) but present relatively less within cytoplasm (Figure 6B, arrowhead). In contrast to Du145-Mock cells, cofilin was distributed across the cytoplasm but not enriched at the cell periphery in Du145-KAI1/CD82 cells (Figure 6B). The phosphorylated or inactivated cofilin was present in the cytoplasm, and there was no obvious difference in subcellular distribution of phosphorylated/inactivated cofilin between Mock and KAI1/CD82 cells (Figure 6C). Thus, although cofilin was still translocated to the peripheral cytoplasm in Du145-KAI1/CD82 cells, KAI1/CD82 expression inhibited the translocation of cofilin to and/or the enrichment of cofilin at the cell periphery, implying a putative mechanism by which Du145-KAI1/CD82 cells fail to form lamellipodia.

We also analyzed the cellular localization of Arp2/3 complex, a downstream effector of Cdc42 and Rac1. Arp2/3 complex plays pivotal roles in nucleation and polymerization of actin [66], especially for the branched cortical actin network [67] at the leading edge of lamellipodia [68]. By analyzing p34, a component of Arp2/3 complex, we found that Arp2/3 complex remained unchanged in both subcellular localization and protein level between Mock and KAI1/CD82 cells (Figure S6, panels A and B). Since N-WASP activates Arp2/3 complex, we examined the effect of N-WASP on actin network. The overexpression of GFP-N-WASP did not rescue the defective actin organization induced by KAI1/CD82 (Figure S6, panel C), as predicted.

**Figure 7:**
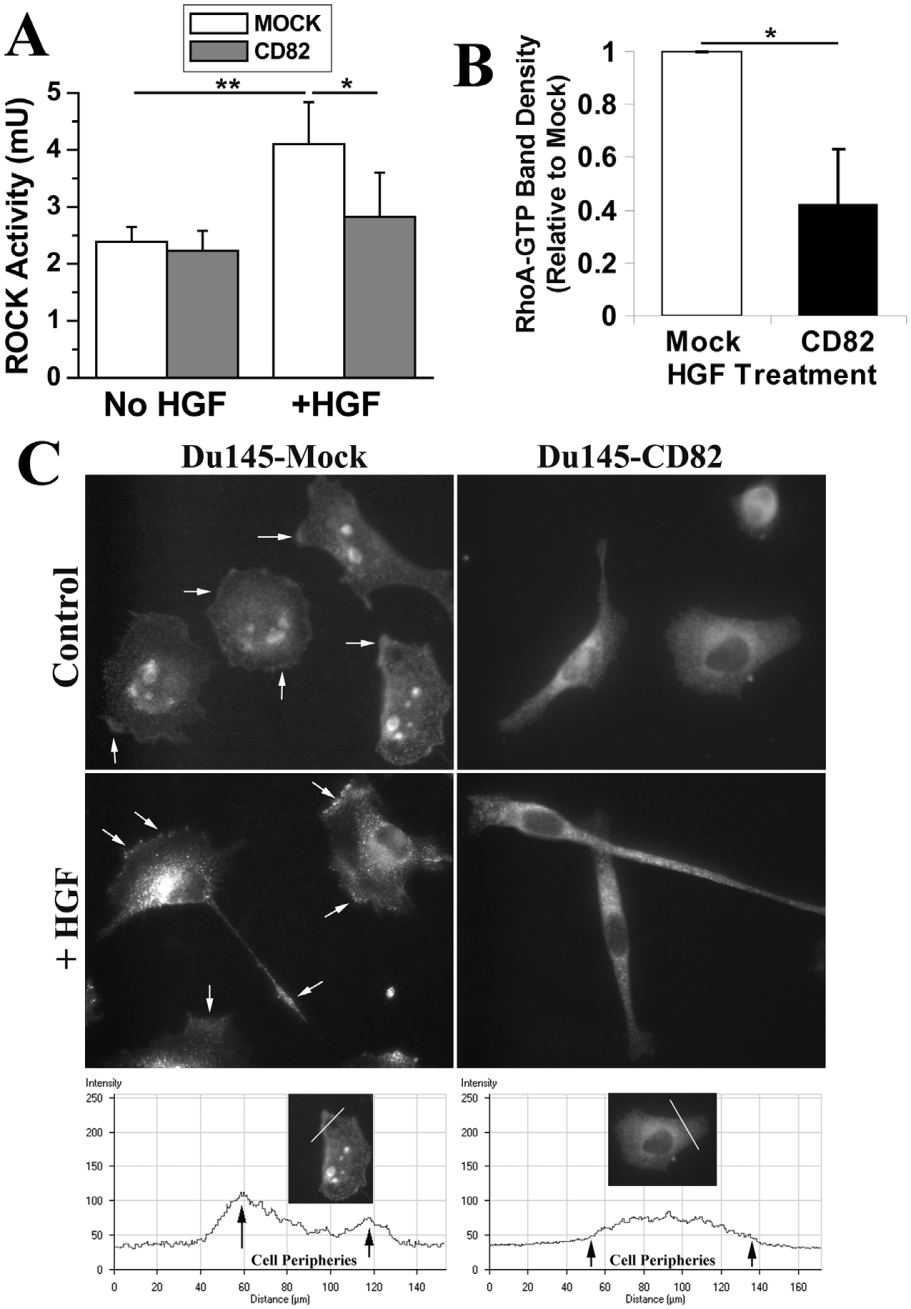
KAI1/CD82 inhibits ROCK activity and the enrichment of RhoA at the cell periphery. (***A***). Du145-Mock and -KAI1/CD82 transfectant cells were cultured in either DMEM containing 0.5% FBS for 40 h or DMEM containing 10% FBS and HGF (100 ng/ml) for overnight. The cells were then lysed with 1% Triton X-100 lysis buffer. Equal amounts of cell lysate were assayed for ROCK activity with MBL Rho Kinase Assay kit. In each experiment, ROCK activities were measured in duplicate for each transfectant. The results shown in histogram are the average values of three individual experiments±SD. *: *P*<0.05, **: *P*<0.01. (***B***). The cells were pretreated with HGF (100 ng/ml) overnight and then assayed for RhoA activity as aforementioned. Histogram represents the relative density of the RhoA-GTP bands (mean±SD, n = 3). *: *P*<0.05. (***C***). The cells spread on LN1 (20 µg/ml)-coated plates were treated with or without HGF (100 ng/ml) overnight and then fixed, permeabilized, and incubated sequentially with RhoA mAb and Alexa594-conjugated second Ab. Images were acquired as described above under a fluorescent microscope. Arrows indicate the enrichment of RhoA at the cell periphery and in the retraction tail. The bottom panel shows the fluorescent profiles of line scan from Mock and KAI1/CD82 cells.

As an effector of RhoA, ROCK is required for cellular retraction by directly or indirectly enhancing the phosphorylation of myosin light chain and consequently increasing the cell contractility [16]. To address the paradox of unchanged RhoA activity and diminished cellular retraction process in KAI1/CD82-expressing cells, we measured the ROCK activity. ROCK activities were equivalent between Du145-Mock and -KAI1/CD82 cells under either regular culture (data not shown) or serum-starved condition (Figure 7A). When cells were treated with HGF, which augments the retraction deficiency in KAI1/CD82 cells (Figure 1C), we found substantial upregulation of ROCK activity in Mock cells but not in KAI1/CD82 cells (Figure 7A), suggesting that KAI1/CD82 significantly suppresses the HGF-induced retraction force. Because RhoA controls ROCK activity, we then analyzed the effect of HGF on RhoA activity. Upon HGF treatment, RhoA activity was markedly enhanced in Mock cells (data not shown) and became much higher in Mock than in KAI1/CD82 cells (Figure 7B). To assess why HGF fails to significantly enhance RhoA activity in Du145-KAI1/CD82 cells, we further assessed the subcellular localization of RhoA to determine whether it can reach to the plasma membrane to be activated. Indeed, RhoA signal could be frequently detected and enriched at the cell periphery, i.e., the plasma membrane, of Mock cells under immunofluorescence, evidenced by the line scan fluorescence profile analysis (Figure 7C). For example, approximately 49% of Mock cells exhibited RhoA enrichment at the cell periphery on LN1-coated plates. But in KAI1/CD82 cells, RhoA was typically diffused cross cytoplasma with a higher concentration in the perinuclear area (Figure 7C). On LN1-coated plates, RhoA was found at the cell periphery in only approximately 11% of KAI1/CD82 cells (Student’s *t*-test, n = 3, p<0.01). The same difference in RhoA distribution between Mock and KAI1/CD82 cells was also observed when the cells were spread on FN-coated substratum (data not shown). Interestingly, RhoA was also localized in the large intracellular vesicles in many Mock cells, but such a localization of RhoA was barely seen in KAI1/CD82 cells. HGF stimulation basically did not alter the difference in RhoA localization, but the intracellular vesicular RhoA in Mock cells disappeared upon HGF stimulation (Figure 7C).

We also examined the formation and maturation of focal adhesions, in which actin fibers anchored to clustered cell-matrix adhesion molecules. Upon KAI1/CD82 overexpression, both matured focal adhesions, revealed by vinculin staining, and immature focal adhesion or focal complexes, revealed by paxillin staining, became grossly diminished (Figure S7).

### KAI1/CD82 Alters the Plasma Membrane PIP_2_, which Directly Triggers Actin Polymerization

**Figure 8:**
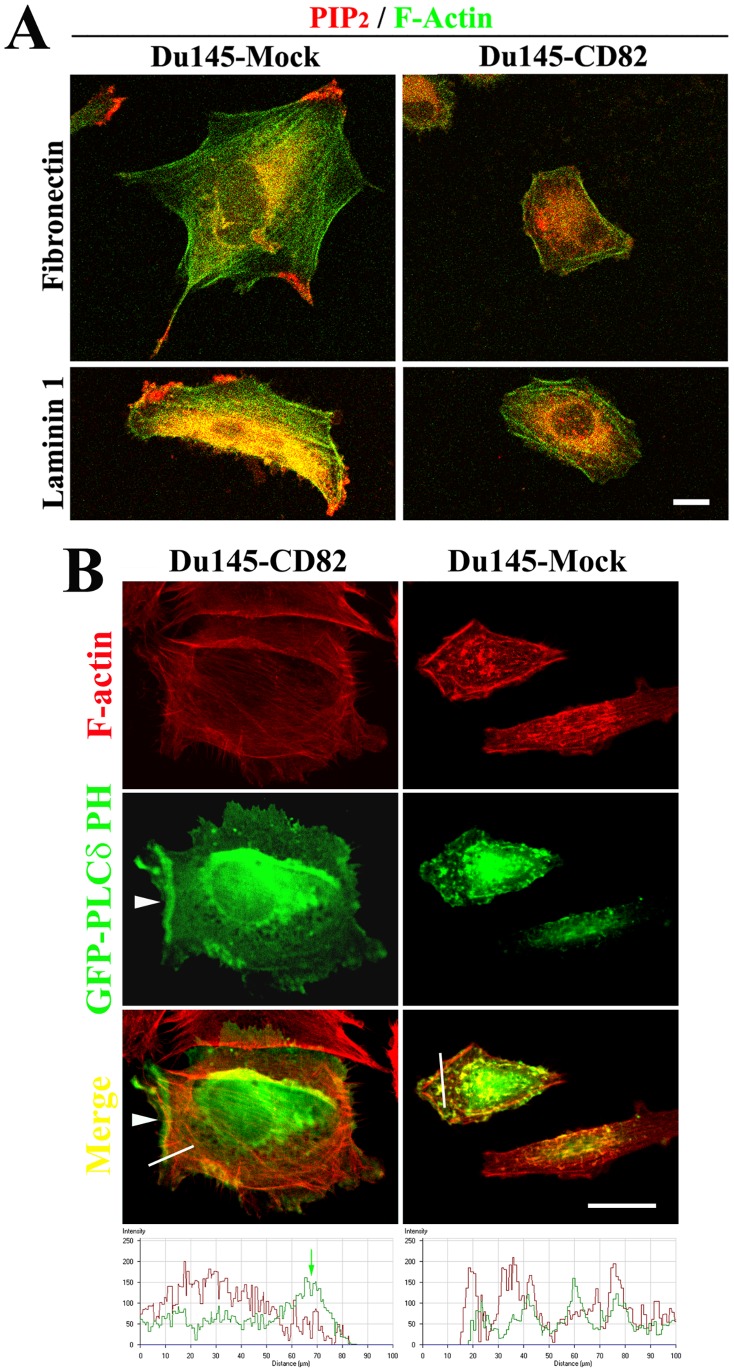
The effect of KAI1/CD82 on the PIP_2_ in the plasma membrane. (***A***). Du145-Mock and -KAI1/CD82 transfectant cells were spread on either FN- or LN1-coated plate in complete DMEM at 37°C overnight, fixed, permebilized, and then incubated with PIP_2_ mAb, followed with the incubation of Alexa 594-conjugated second Ab and FITC-conjugated phalloidin. Confocal images of X-Y sections were collected, scale bar: 20 µm. (***B***). The pEGFP-PLCδ PH domain construct was transiently transfected into Du145-Mock and -KAI1/CD82 transfectants. At 48 h after transfection, the cells were spread on an FN (10 µg/ml)-coated plate, fixed, permeabilized, stained with Alexa 594-conjugated phalloidin, and analyzed with confocal microscopy. The X-Y section images were captured as described above. Arrowhead: the localization of EGFP-PLCδ PH domain at the cell periphery. Scale bar: 20 µm. The bottom panel shows F-actin (red) and PLCδ PH domain (green) fluorescent profiles of line scan (white lines in the Merge images) from Mock and KAI1/CD82 cells. Green arrow: the enrichment of PLCδ PH domain at the cell periphery.

The facts of diminished enrichment of cofilin at the periphery of KAI1/CD82-expressing cells and less integrin-dependent actin polymerization drove us to analyze the cellular distribution of PIP_2_, an anchoring site of cofilin and initiator of actin polymerization at the plasma membrane [69], [70]. The localization of PIP_2_, probed by a mAb specifically against PIP_2_, was found at the cell periphery such as the leading edge of lamellipodia, end of filapodia, and trailing edge of the retraction tail in Du145-Mock cells spread on FN or LN 1 (Figure 8A). In Du145-CD82 cells, however, the mAb-detectable PIP_2_ was barely localized at the cell periphery. The staining patterns of PIP_2_ Ab were consistent with the one of cofilin. When probed by GFP-PLCδ PH domain fusion, which specifically binds to the membrane PIP_2_, PIP_2_, reflected by the GFP signals, was more concentrated at the plasma membrane in Mock cells but more localized in the cytoplasmic area in CD82 cells (Figure 8B). The localization of PIP_2_ at the cell periphery or plasma membrane was constantly observed in forms of continuous lines (arrowhead), thick patches (not shown), or large clusters (not shown) in Mock cells. But in CD82 cells, less PIP_2_ was found at the cell periphery, and even there PIP_2_ more often exhibited as dots or small patches. Selective enrichment of GFP-PLCδ PH domain at the cell periphery of Mock cells was supported by the line scan fluorescence profile analysis. Hence the results from PIP_2_ mAb and GFP-PLCδ PH domain agree with each other.

## Discussion

We herein demonstrated that, as a tumor metastasis suppressor, KAI1/CD82 inhibits cell movement by attenuating the formation and development of both cellular protrusion and retraction at the cellular level (Figure S8). At the molecular level, such attenuations result from the deficiency in the plasma membrane-initiated assembly and organization of actin cytoskeleton (Figure S8). From signaling point of view, KAI1/CD82 deregulates the activities of Rac1, RhoA, and their effectors through perturbing multiple signaling pathways. (Figure S8).

### Alterations in Cellular Morphology upon KAI1/CD82 Expression Reflect the Deficiencies in Protrusion and Retraction - the Motility-inhibitory Mechanism at the Cellular Level

KAI1/CD82 expression alters cellular morphology in various aspects. These alterations result from and reflect both diminished protrusive activity, e.g., the reduced formation of lamellipodia, and attenuated retraction activity, e.g., the elongated trailing tail. Because migrating cells typically display these morphological changes, loss or disruption of these motility-related events is therefore likely to be important for the cell movement-inhibitory activity of KAI1/CD82.

In Du145 prostate cancer cells, KAI1/CD82 expression abolished the lamellipodia formation on FN-coated plates or regular cell culture flask. In PC3 prostate cancer cells, KAI1/CD82 inhibited lamellipodia formation extensively on FN but partially on LN. Because PC3 cells form fan-like, well-developed lamellipodia on LN, KAI1/CD82 more likely inhibits the development or prevailing of fan-like lamellipodia. Of note, the definition of lamellipodia herein covers both recently designated lamellipodia and lamella [71]. Because both Du145 and PC3 cells form lamellipodia during cell migration, the lack or disruption of lamellipodia formation or development is very likely crucial for the motility-inhibitory activity of KAI1/CD82.

The presence of elongated cellular extensions is another morphological alteration of KAI1/CD82 overexpression in Du145 and PC3 cells. The elongated cellular extensions could be the consequence of excessive protrusive activity or could also reflect retraction deficiency. Our study demonstrated that various cellular extensions in KAI1/CD82-expressing Du145 and PC3 cells were caused by deficiency in the retraction process. Earlier studies showed that the immobilized-KAI1/CD82 mAbs induce profound dendritic extensions in hematopoetic cells [12], [15], morphologically reminiscent of some types of cellular extensions seen in KAI1/CD82-expressing Du145 and PC3 cells. Despite the differences between overexpression and Ab engagement and between adherent and suspension cells, these dendritic extensions more likely represent the phenotype of less retraction if the motility-inhibitory function of KAI1/CD82 is considered.

KAI1/CD82 blocked not only the cellular protrusion and retraction processes that were induced by the integrin-ECM engagement but also these events stimulated by growth factors or serum (Figure 1). For example, KAI1/CD82-caused deficiency in real tail retraction was manifested in the presence of HGF. These observations indicate that KAI1/CD82 inhibits both integrin and growth factor signaling. Another novel observation in this study is that the effect of KAI1/CD82 on these motility-required cellular processes appeared to be independent of cell-cell contacts. In other words, KAI1/CD82 can induce aforementioned morphological changes without directly engaging a cellular receptor from adjacent cells, because those morphological effects are pronounced when cells do not form cell-cell contacts. On the contrary, cell-cell contacts alleviate the effect of KAI1/CD82 on actin cytoskeleton organization, which determines cellular morphological changes.

### KAI1/CD82-induced Deficiencies in Protrusion and Retraction Processes are caused by Aberrant Actin Organization and Polymerization

The profound morphological changes induced by KAI1/CD82 apparently resulted from the aberrant organization and/or reorganization of cellular cytoskeleton networks. Actin is the cytoskeleton system that drives the cell movement-related subcellular events including protrusion, traction, and retraction [39], [72]–[76]. As predicted, actin cytoskeleton became globally aberrant upon KAI1/CD82 overexpression. The reduced or deficient cortical meshwork and stress fibers in KAI1/CD82-overexpressing cells suggested the aberrancy in actin polymerization. Such aberrancy can be further exacerbated by the reduced integrin signaling in KAI1/CD82-overexpressing cells.

The development of cortical actin network generates protrusive force, morphologically revealed as the lamellipodia formation [39]. Therefore, the formation of actin cortical meshwork and simultaneous extension of leading lamellipodia are the major subcellular morphological features of many migrating cells. In KAI1/CD82-overexpressing Du145 cells, the loss of these morphological characteristics was apparently caused by aberrant actin organization, particularly the polymerization of branched, cortical actin meshwork.

In parallel, the retraction process is also essential for the movement of many types of cells [39]. The defects in retraction upon KAI1/CD82 expression were displayed as either elongated trailing tails when Du145-KAI1/CD82 cells were treated with HGF or spread on LN1-coated plates, long cellular extensions in the cells with bipolar or dumbbell shape, or persistent vertices in cells with irregular polygonal shape. Mechanistically, the deficiency in retraction could result from an abnormality of actin-myosin retraction machinery. In the case of KAI1/CD82, the decreased ROCK activity caused functional incompetence of this machinery while the fewer stress fibers made the retraction process lose its structural base.

### Aberrant Actin Cytoskeleton in KAI1/CD82-expressing Cells Results from the Deregulation of Rac, Rho, and their Effectors - the Molecular Mechanism

Rho small GTPases are the master regulators of actin reorganization. Rac activation stimulates membrane ruffling through polymerization of cortical actin near the cell periphery, while Rho activation stimulates cell contractility through assembly of mainly radial-oriented actin stress fibers [16], [77]. Our study revealed that the aberrant cytoskeleton reorganization upon KAI1/CD82 expression resulted from the imbalance of Rho GTPase activities. Such a defect in cytoskeletal reorganization effects mainly on cell adhesion proteins because they are directly connected to cytoskeleton. For example, the reduced cytoskeletal reorganization will weaken the cytoskeletal engagement of integrins in tetraspanin-enriched microdomains (TEMs) and subsequently lead to the local attenuation of integrin signaling or even inactivation of integrins. We extrapolate that both imbalance of Rho GTPases and inactivation of integrins result in the aberrancy in cellular morphology and diminishment in cell motility.

Consistent with the current understanding of the roles of Rac in cell morphology and movement, the suppressed lamellipodia and cell movement upon KAI1/CD2 expression correlated well with the diminished Rac1 activity in Du145 cells.

In some cell types, Rac1 activation negatively regulates RhoA activity by generating reactive oxygen species and subsequently activating p190RhoGAP at the plasma membrane [64]. The delicate balance between the antagonistic activities of Rac1 and RhoA is crucial for proper cell movement and also specifies cell morphology [64], [78]. In addition, KAI1/CD82 was reported to inhibit the activity of Src kinases [42], which activates p190RhoGAP by tyrosine phosphorylation [64], and the lower Src activity ultimately leads to the RhoA activation. However, the constitutive RhoA activity appears to be unaltered upon KAI1/CD82 expression. Although HGF markedly enhanced RhoA activity, KAI1/CD82 apparently could block such stimulation, reflected by the defect in retraction and the formation of fewer stress fibers in KAI1/CD82-expressing cells. It remains unclear that, in Du145-KAI1/CD82 cells, RhoA still exhibits a similar level of constitutive activity, although it cannot reach the plasma membrane.

As a key effector of both Rac and Rho, cofilin plays an important role in membrane ruffling [79] or lamellipodia formation [16], [80]–[84]. Driven by activated Rac or PIP_2_, cofilin is translocated to or enriched in the cell periphery [85]–[88] where it interacts with actin cytoskeleton, generates more barbed ends, and promotes actin cortical meshwork formation and consequently lamellipodia formation [16]. Translocation of cofilin to the plasma membrane is considered to be an indicator of cofilin activation [85]–[88]. Interestingly, the subcellular localizations of total and inactivated cofilin in Du145-Mock and -KAI1/CD82 cells displayed distribution patterns similar to the ones in migrating and nonmigrating fibroblast cells [69], respectively. Upon KAI1/CD82 expression, cofilin was no longer enriched at the cell periphery, although it could translocate to the peripheral cytoplasm. This observation strongly suggests that KAI1/CD82 expression blocks the translocation and therefore activation of cofilin, underlining a mechanism by which KAI1/CD82 impairs lamellipodia formation. If so, one would expect more phosphorylated or inactive cofilin in Du145-KAI1/CD82 cells. However, the level of phosphorylated cofilin proteins remained unchanged upon KAI1/CD82 expression. Possibly, cofilin in Du145-KAI1/CD82 cells can still undergo dephosphorylation during translocation to the peripheral cytoplasm but cannot become enriched at the cell periphery due to aberrant plasma membrane in Du145-KAI1/CD82 cells.

When KAI1/CD82 is present, HGF cannot upregulate the activities of ROCK and its direct upstream activator RhoA. Besides agreeing with earlier observations that KAI1/CD82 inhibits HGF/c-Met signaling [41], [42], [56], these findings have further demonstrated that CD82 intercepts the HGF/c-Met signaling leading to the cellular retraction. Because KAI1/CD82 can inhibit the retraction process even without HGF stimulation, we predict that the local, constitutive ROCK activity at the retraction areas in KAI1/CD82-expressing cells is lower but the assay for ROCK activity may not be sensitive enough to report the local diminution of ROCK activity.

In addition to deregulating Rho GTPases and their effectors, KAI1/CD82 may also affect the trafficking of actin-binding proteins that are associated with TEMs to alter actin organization, based on the early observations that KAI1/CD82 proteins traffic between the plasma membrane and endosomes/lysosmes [89] and regulate the trafficking of their associated integrins [8].

### KAI1/CD82 Intercepts Multiple Signaling Pathways: a Problem of the Plasma Membrane?

As described earlier, KAI1/CD82 can initiate outside-in signaling [13], [15], [58]. KAI1/CD82-initiated signaling may intercept the promigration signaling derived from integrin and growth factor receptor. Alternatively, because KAI1/CD82 physically interacts with β1 integrins and growth factor receptors and downregulates their function, KAI1/CD82 can directly inhibit the promigration signaling at the very upstream. If the complexity of tetraspanin-enriched microdomain (TEM) constituents is taken into consideration, multiple signaling pathways are susceptible to KAI1/CD82 inhibition. Indeed, we found in this study that KAI1/CD82-induced morphological and cytoskeletal changes could not simply be overridden or bypassed by one or two signaling mechanisms. For example, the signaling originating from β1 integrin [39], EGFR [40], c-Met [41], [42], [90]–[92], and CXCR4 [93]–[97] promotes cell migration and actin reorganization through Rho small GTPases. Activation of these signaling pathways either alone or together, however, cannot reverse KAI1/CD82-induced morphological and cytoskeletal effects. This observation strongly suggests that KAI1/CD82 acts either on the signaling step(s) at or after the converge point(s) of multiple pathways, e.g., the signaling that immediately leads to actin reorganization, or on the very beginning of multiple signaling pathways, e.g., TEMs, in which integrins and growth factor receptors reside. In either case, KAI1/CD82 likely acts directly at the plasma membrane where actin reorganization is triggered during cell migration and TEMs are located.

The plasma membrane PIP_2_ is apparently perturbed by KAI1/CD82 overexpression. Because the PIP_2_ mAb probes PIP_2_ after the fixation procedure, it can detect only free PIP_2_ at the plasma membrane. While GFP-PLCδ PH domain fusion not only binds free PIP_2_ but also likely competes with endogenous PIP_2_-binding proteins in live cells to occupy PIP_2_. Thus, the readout of GFP-PLCδ PH domain fusion more likely reflects the level of free and occupied PIP_2_ at the plasma membrane. In either case, more PIP_2_ was found in the plasma membrane of Mock cells. PIP_2_ links the plasma membrane to actin cytoskeleton by either directly binding and/or activating actin-binding proteins such as β-spectrin, α-actinin, vinculin, and ERM proteins or indirectly inducing the cortical actin polymerization through profilin, cofilin, and N-WASP [70], [98]. The level of PIP_2_ controls the connection of the membrane lipid bilayer to its underlying actin cytoskeleton [99]. The reduced PIP_2_ at the plasma membrane upon KAI1/CD82 expression, especially at the membrane sites where actin actively undergoes reorganization, likely causes attenuated actin polymerization during cell spreading and migration. Moreover, the composition and distribution of membrane lipid also affect the functional status of Rho GTPases and their effectors because, to be functional, Rho GTPases and their effectors must translocate to the plasma membrane [100]. Because KAI1/CD82 regulates the composition and distribution of PIP_2_ (this study) and other lipids [41], [101] at the plasma membrane, it likely alters the activities of Rho GTPases and their effectors through the translocation step.

Interestingly and also surprisingly, cell-cell adhesion significantly alleviates the morphological and cytoskeletal effects of KAI1/CD82. Although KAI1/CD82 has been reported to upregulate cell-cell adhesion or aggregation [10], [102]–[105], associate with E-cadherin in colon cancer cells [106], and bind to a counter-receptor DARC [107], how KAI1/CD82 regulates cell-cell adhesion is poorly understood. Likely, cell-cell adhesion corrects, to some degree, the imbalanced Rac and Rho activities in Du145-KAI1/CD82 cells as cell-cell contacts typically promote Rac activity and cortical actin network formation but suppress Rho activity and stress fiber formation. Hence, KAI1/CD82 likely induces the motility-related morphological and cytoskeletal phenotypes 1) only when cell-cell adhesion falls apart and 2) through cell-ECM adhesion. In other words, KAI1/CD82 probably inhibits only the movement of isolated individual cells.

In conclusion, we demonstrated in this study that KAI1/CD82 likely intercepts multiple signaling pathways at the plasma membrane by perturbing membrane lipids. This perturbation results in the imbalance of Rac, Rho, and their effector activities and the aberrancy in actin organization and reorganization. The cytoskeletal abnormality in KAI1/CD82-expressing cancer cells causes the attenuation of both cellular protrusion and retraction processes, which ultimately leads to the suppression of cell movement (Figure S8). Although tetraspanins physically associate with each other and functionally crosstalk, each tetraspanin has specific functional identity, evidenced by the unique phenotypes displayed by the knockout mouse lines of several tetraspanins. Also, our earlier study underpinned that CD82-mediated inhibition of cell movement is independent of other tetraspanins [108]. Thus, the motility-inhibitory mechanism described above appears to be specific to KAI1/CD82.

## Supporting Information

Figure S1 **Illustration of the F-actin quantification in subcellular regions.**
(TIF)

Figure S2 **The levels of KAI1/CD82 in Du145 transfectant cells and PrECs.** Du145-Mock and -KAI1/CD82 transfectant cells and PrECs were lysed in RIPA cell lysis buffer and examined by Western blot with KAI1/CD82 mAb M104 (top panel) or actin Ab (bottom panel).
(TIF)

Figure S3 **Morphological phenotypes of KAI1/CD82-overexpressing PC3 cells.** (***A***) KAI1/CD82 expression altered cell morphology. PC3-Mock and -KAI1/CD82 transfectant cells were plated on tissue culture flasks at 37°C, 5% CO_2_ overnight in DMEM medium containing 10% FCS. (***B***) Diminished lamellipodia and elongated extension in KAI1/CD82-overexpressing PC3 cells. PC3-Mock and -KAI1/CD82 transfectant cells were spread on FN- or LN1-coated plates and stained with tetraspanin CD81 mAb M38 using immunofluorescence as described [55] to visualize cell peripheries. Arrow indicates the elongated cellular extension.
(TIF)

Figure S4 **The actin cortical meshwork and stress fiber were disrupted upon KAI1/CD82 expression in PC3 cells.** After being spread on FN (50 µg/ml)- or LN1 (50 µg/ml)-coated coverslips in serum-free DMEM at 37°C, 5% CO_2_ for 6 h, PC3 transfectant cells were fixed, permeabilized, and then stained with TRITC-conjugated α-phalloidin. The fluorescent images were captured under an Axiophot fluorescent microscope equipped with an Optronics digital camera at magnification 63X.
(TIF)

Figure S5 **The effect of cell density on the CD81-CD82 association.** Du145-CD82 transfectant cells were grown in complete media to either confluence (the “dense” condition) or 50% confluence (the “sparse” condition) and lysed with 1% Brij 98 lysis buffer. The cell lysates were incubated with either CD81 mAb M38 or control IgG, and the immunoprecipitates and lysates were blotted with CD82 mAb TS82b after SDS-PAGE separation and electric transferring. The CD81-coprecipitated CD82 proteins were quantified with densitometry analysis, and the results were normalized by total cellular CD82 and presented as relative density to the sparse condition (mean±SD, n = 3).
(TIF)

Figure S6 **KAI1/CD82 does not alter the protein level and subcellular localization of the p34 protein in Arp2/3 complex.** *(* ***A*** *)* The p34 protein levels in Du145-Mock and -KAI1/CD82 cells were assessed by Western blot. Tubulin blot is used as a control for protein loading. *(* ***B*** *)* Du145 transfectant cells were spread on FN-coated coverslips in complete DMEM from 3 to 6 h. The cells were fixed, permeabilized, and incubated with p34 pAb and TRITC-conjugated α-phalloidin, followed by the FITC-conjugated second Ab staining. Images were captured under a confocal microscope and each image represents a single X-Y section. Scale bar, 20 µm. *(* ***C*** *)* The pEGFP-WASP construct was transiently transfected into Du145-Mock and -KAI1/CD82 transfectant cells. At 48 h after transfection, the cells were spread on an FN (10 µg/ml)-coated plate, fixed, permeabilized, stained with Alexa 594-conjugated phalloidin, and analyzed with confocal microscopy. Scale bar, 20 µm.
(TIF)

Figure S7 **The effect of KAI1/CD82 overexpression on the formation and maturation of focal adhesion.** Du145-Mock and -KAI1/CD82 transfectant cells were spread on either FN (50 µg/ml)- or LN1(50 µg/ml)-coated plate in complete DMEM at 37°C overnight, fixed, permebilized, and then incubated with vinculin (***A***) or paxillin (***B***) mAb, followed with the incubation of Alexa 594-conjugated second Ab. Images were acquired with fluorescent microscopy.
(TIF)

Figure S8 **The mechanism by which KAI1/CD82 inhibits the movement of tumor cells.**
(TIFF)
