# Supplementary figures and images for: Tetraspanin CD82 Inhibits Protrusion and Retraction in Cell Movement by Attenuating the Plasma Membrane-Dependent Actin Organization
Source: PLoS One. 2012 Dec 14;7(12):e51797. doi: 10.1371/journal.pone.0051797 (PMC3522597; doi:10.1371/journal.pone.0051797)

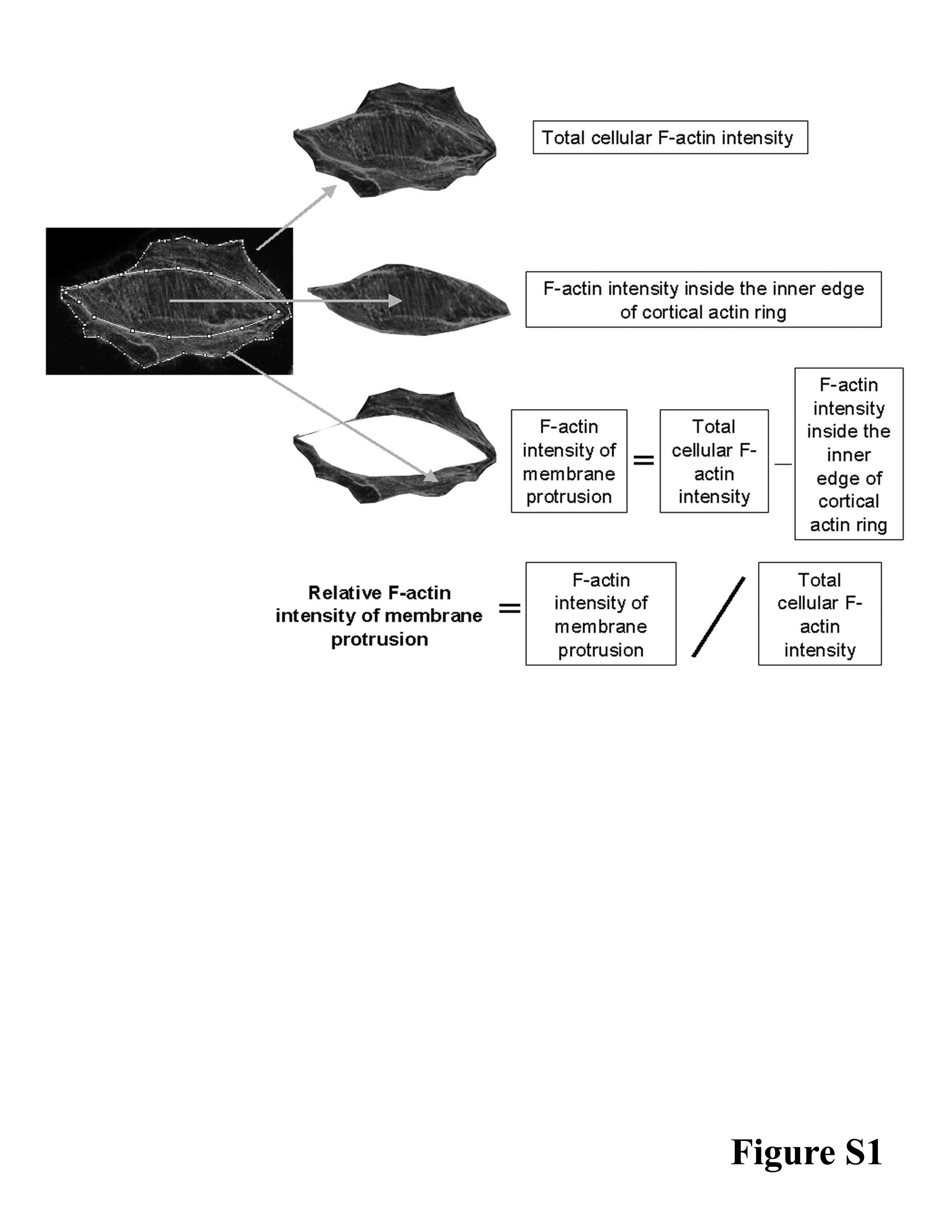

Supplement: Figure S1 — Illustration of the F-actin quantification in subcellular regions. (TIF) [file pone.0051797.s001.tif]

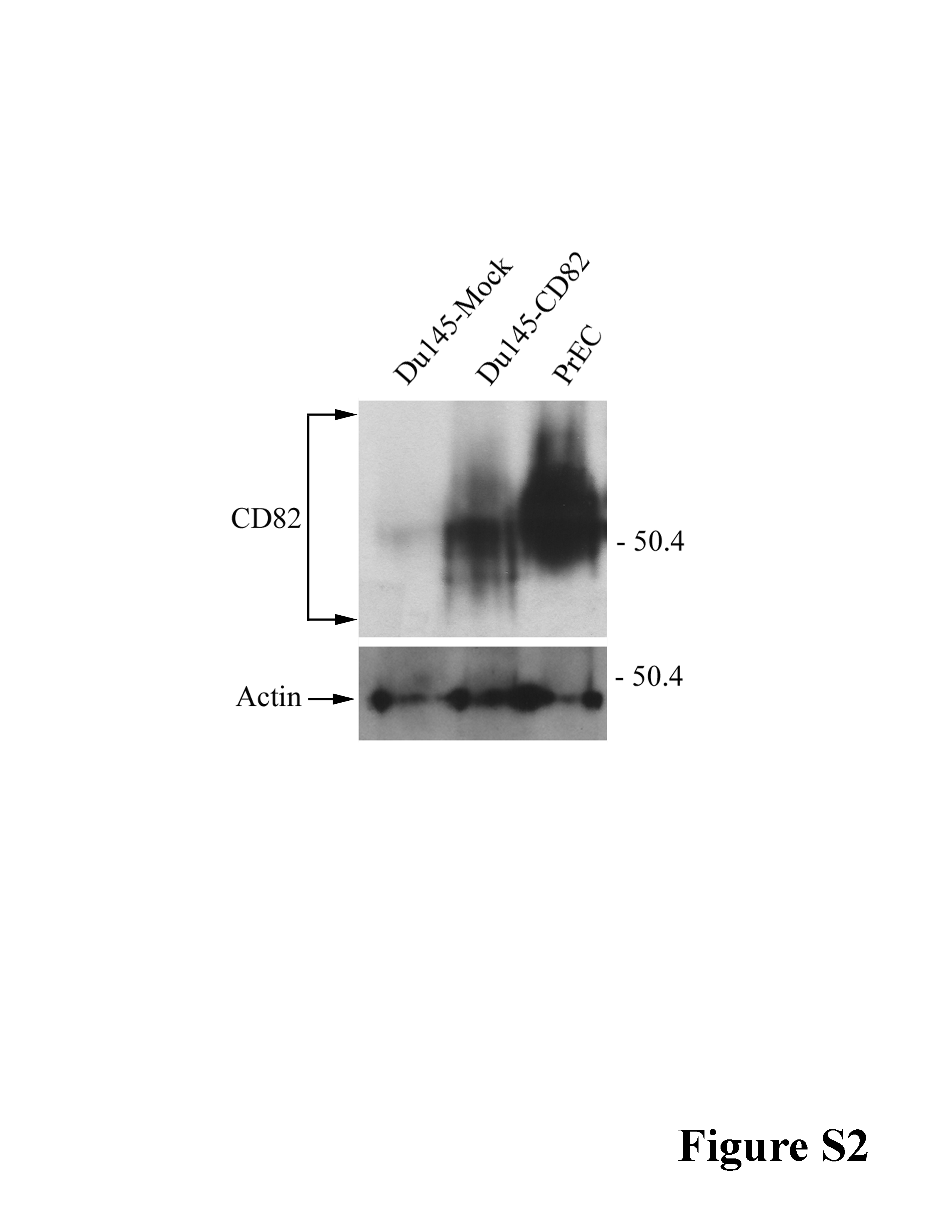

Supplement: Figure S2 — The levels of KAI1/CD82 in Du145 transfectant cells and PrECs. Du145-Mock and -KAI1/CD82 transfectant cells and PrECs were lysed in RIPA cell lysis buffer and examined by Western blot with KAI1/CD82 mAb M104 (top panel) or actin Ab (bottom panel). (TIF) [file pone.0051797.s002.tif]

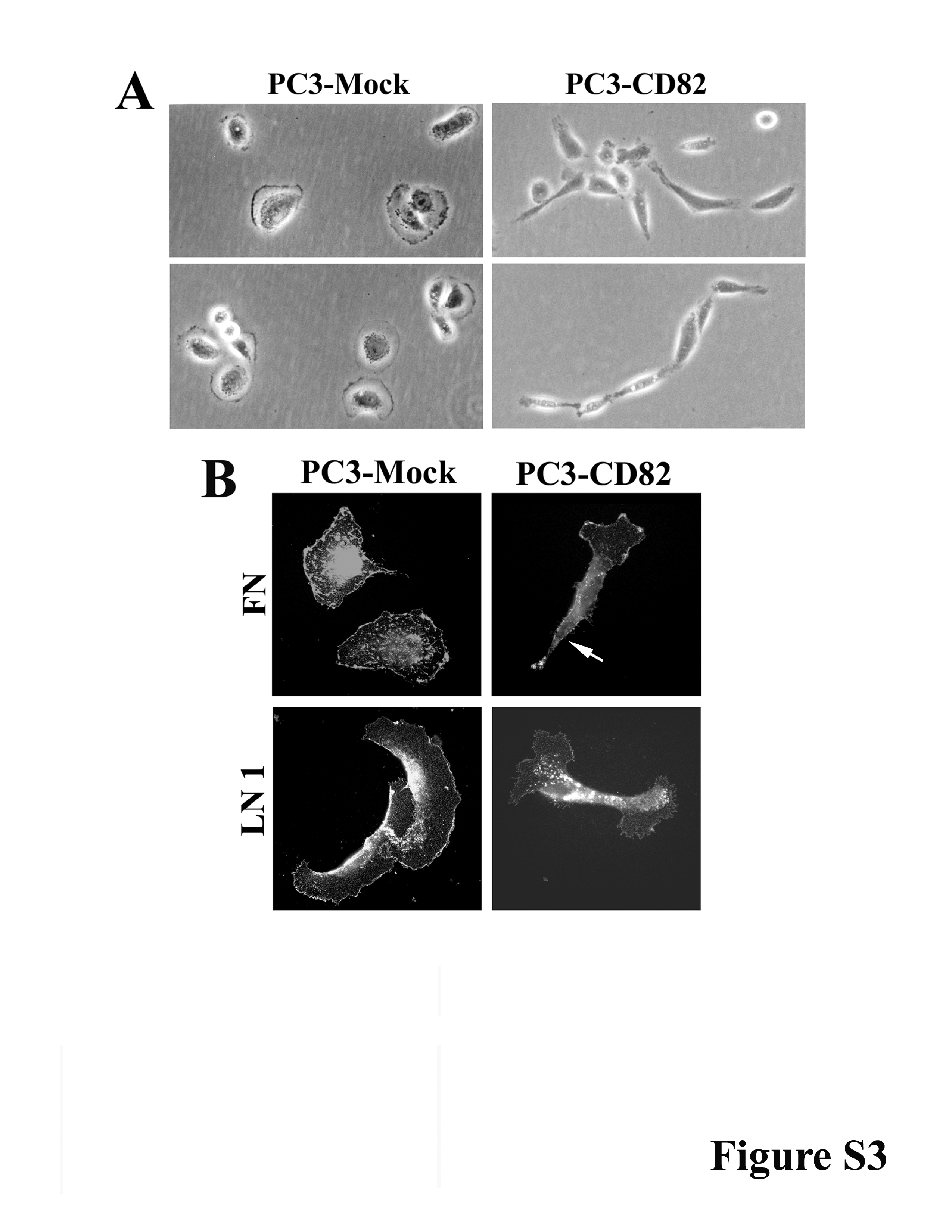

Supplement: Figure S3 — Morphological phenotypes of KAI1/CD82-overexpressing PC3 cells. (A) KAI1/CD82 expression altered cell morphology. PC3-Mock and -KAI1/CD82 transfectant cells were plated on tissue culture flasks at 37°C, 5% CO2 overnight in DMEM medium containing 10% FCS. (B) Diminished lamellipodia and elongated extension in KAI1/CD82-overexpressing PC3 cells. PC3-Mock and -KAI1/CD82 transfectant cells were spread on FN- or LN1-coated plates and stained with tetraspanin CD81 mAb M38 using immunofluorescence as described [55] to visualize cell peripheries. Arrow indicates the elongated cellular extension. (TIF) [file pone.0051797.s003.tif]

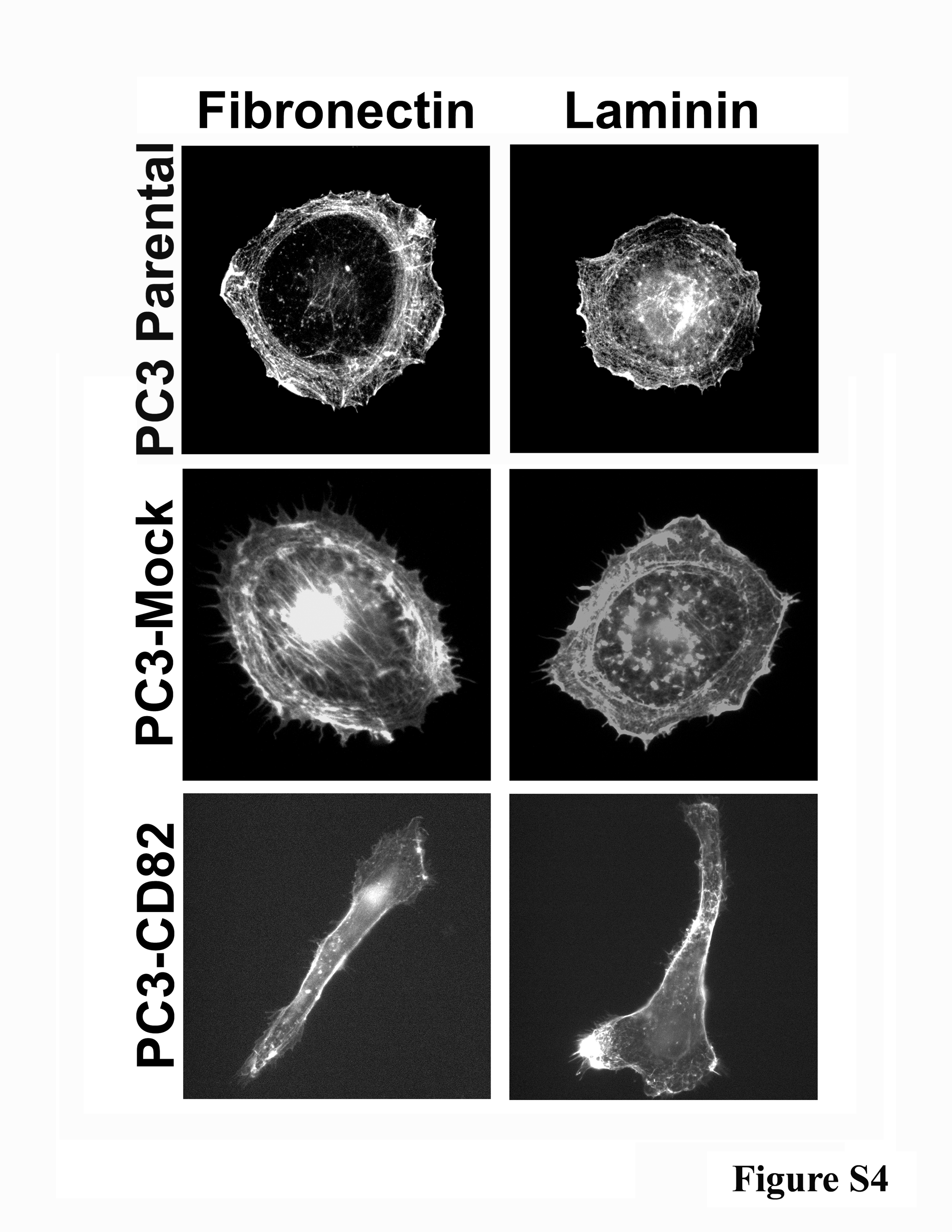

Supplement: Figure S4 — The actin cortical meshwork and stress fiber were disrupted upon KAI1/CD82 expression in PC3 cells. After being spread on FN (50 µg/ml)- or LN1 (50 µg/ml)-coated coverslips in serum-free DMEM at 37°C, 5% CO2 for 6 h, PC3 transfectant cells were fixed, permeabilized, and then stained with TRITC-conjugated α-phalloidin. The fluorescent images were captured under an Axiophot fluorescent microscope equipped with an Optronics digital camera at magnification 63X. (TIF) [file pone.0051797.s004.tif]

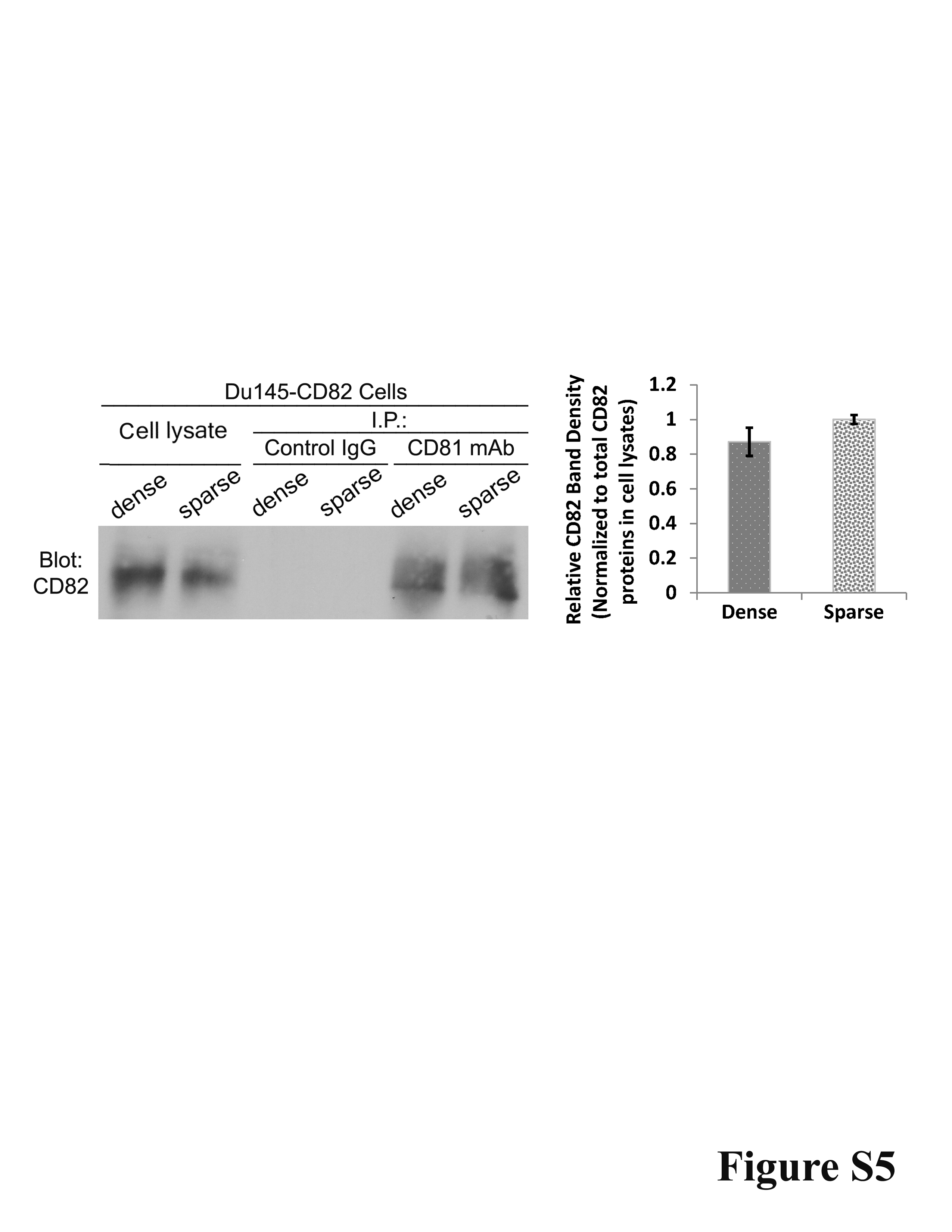

Supplement: Figure S5 — The effect of cell density on the CD81-CD82 association. Du145-CD82 transfectant cells were grown in complete media to either confluence (the “dense” condition) or 50% confluence (the “sparse” condition) and lysed with 1% Brij 98 lysis buffer. The cell lysates were incubated with either CD81 mAb M38 or control IgG, and the immunoprecipitates and lysates were blotted with CD82 mAb TS82b after SDS-PAGE separation and electric transferring. The CD81-coprecipitated CD82 proteins were quantified with densitometry analysis, and the results were normalized by total cellular CD82 and presented as relative density to the sparse condition (mean±SD, n = 3). (TIF) [file pone.0051797.s005.tif]

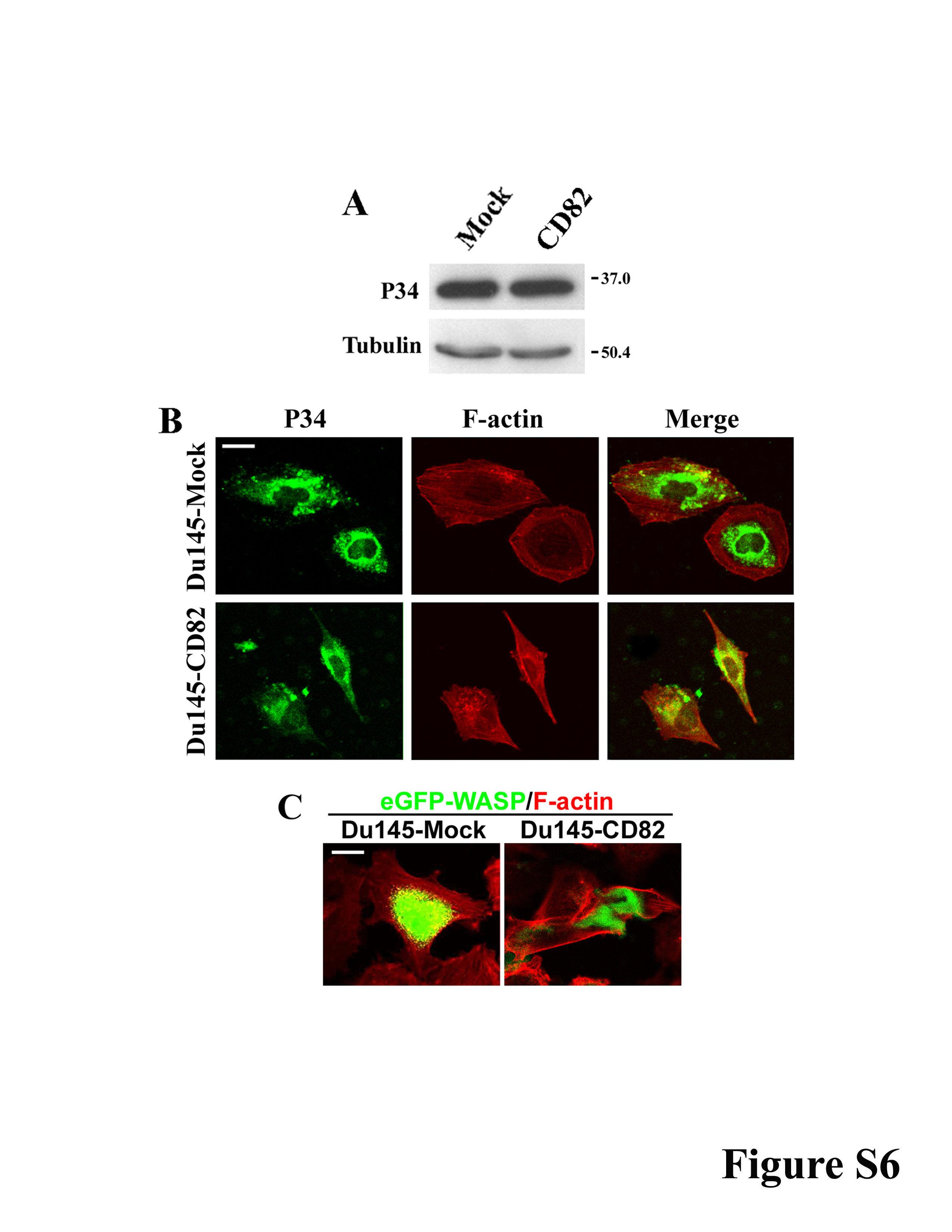

Supplement: Figure S6 — KAI1/CD82 does not alter the protein level and subcellular localization of the p34 protein in Arp2/3 complex. ( A ) The p34 protein levels in Du145-Mock and -KAI1/CD82 cells were assessed by Western blot. Tubulin blot is used as a control for protein loading. ( B ) Du145 transfectant cells were spread on FN-coated coverslips in complete DMEM from 3 to 6 h. The cells were fixed, permeabilized, and incubated with p34 pAb and TRITC-conjugated α-phalloidin, followed by the FITC-conjugated second Ab staining. Images were captured under a confocal microscope and each image represents a single X-Y section. Scale bar, 20 µm. ( C ) The pEGFP-WASP construct was transiently transfected into Du145-Mock and -KAI1/CD82 transfectant cells. At 48 h after transfection, the cells were spread on an FN (10 µg/ml)-coated plate, fixed, permeabilized, stained with Alexa 594-conjugated phalloidin, and analyzed with confocal microscopy. Scale bar, 20 µm. (TIF) [file pone.0051797.s006.tif]

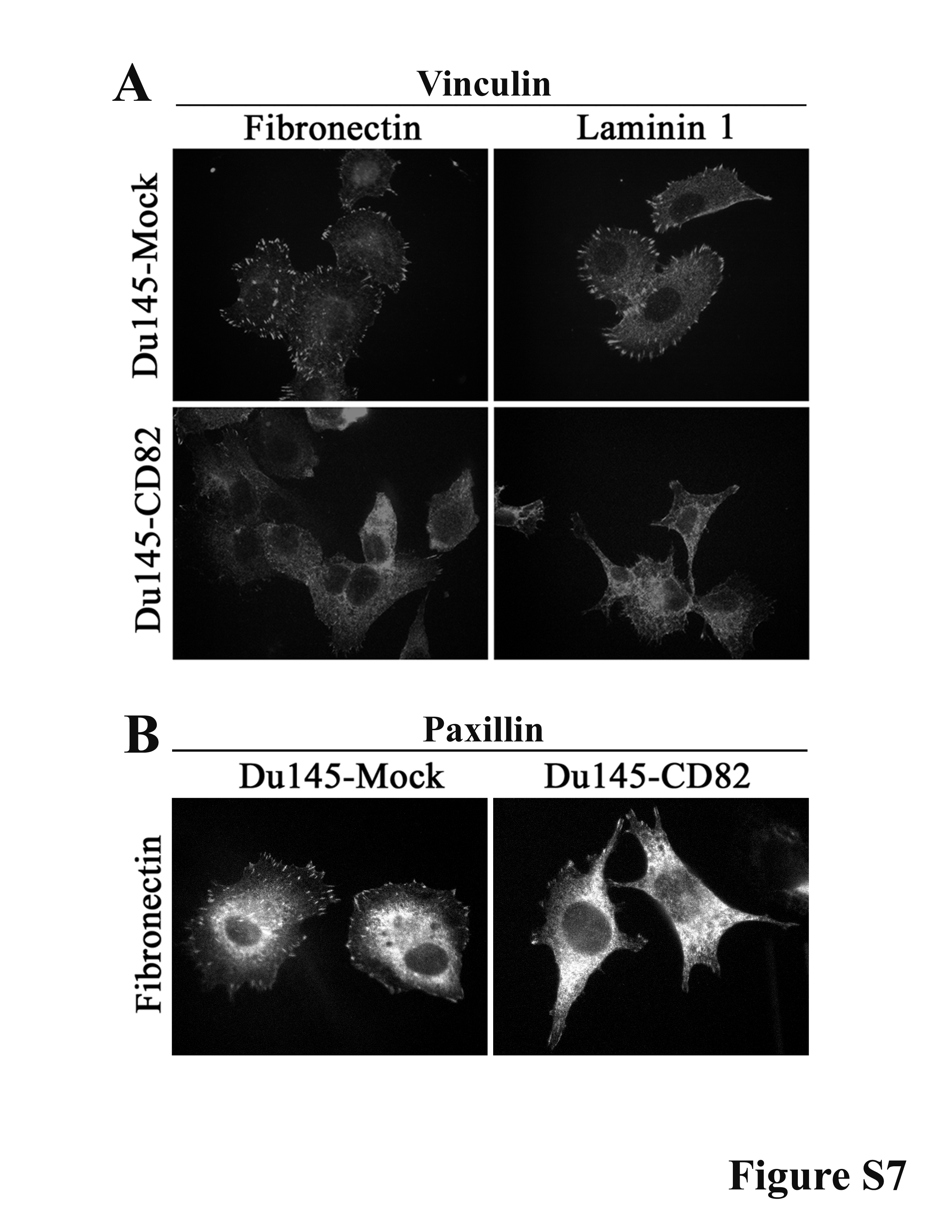

Supplement: Figure S7 — The effect of KAI1/CD82 overexpression on the formation and maturation of focal adhesion. Du145-Mock and -KAI1/CD82 transfectant cells were spread on either FN (50 µg/ml)- or LN1(50 µg/ml)-coated plate in complete DMEM at 37°C overnight, fixed, permebilized, and then incubated with vinculin (A) or paxillin (B) mAb, followed with the incubation of Alexa 594-conjugated second Ab. Images were acquired with fluorescent microscopy. (TIF) [file pone.0051797.s007.tif]

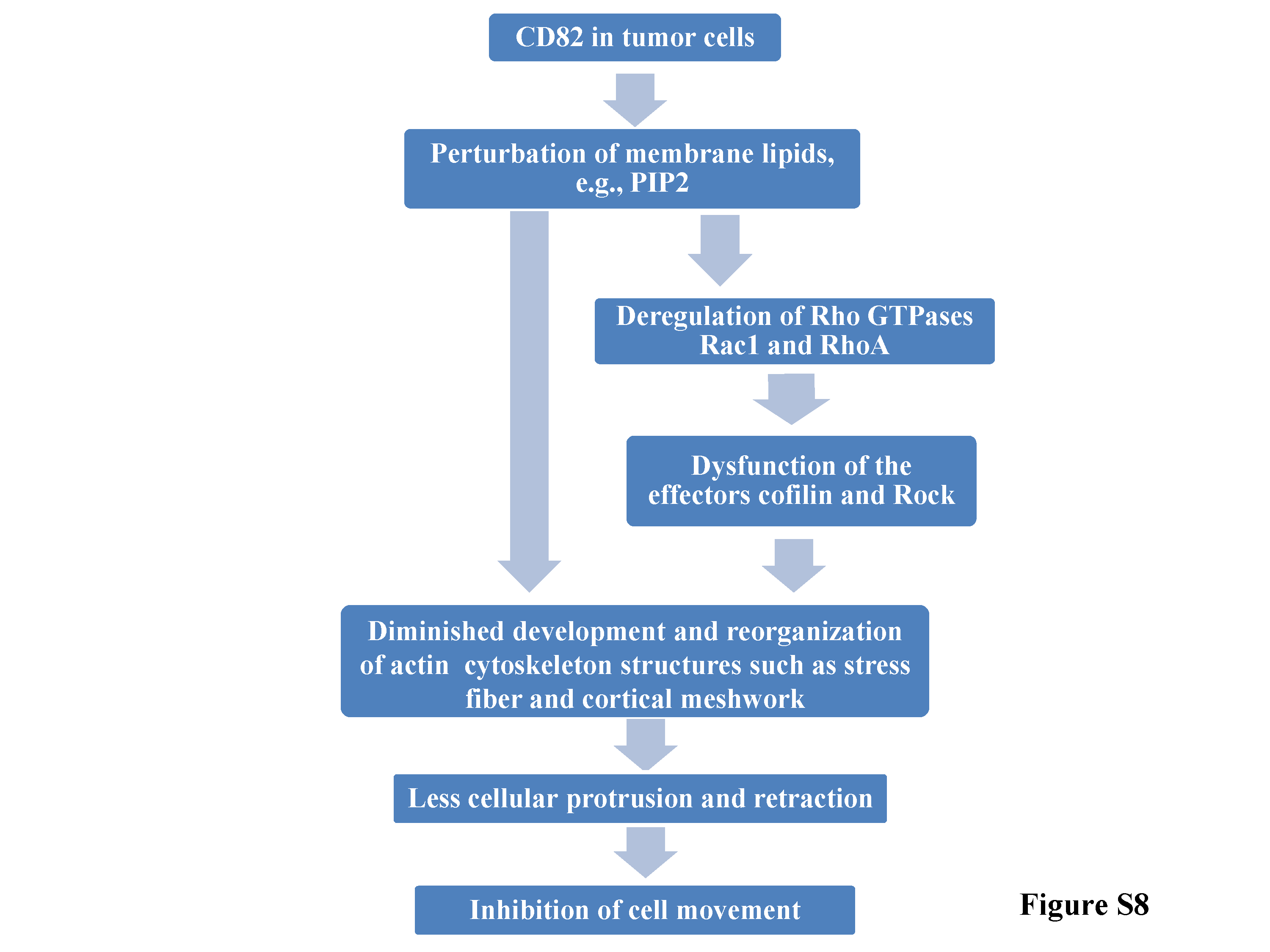

Supplement: Figure S8 — The mechanism by which KAI1/CD82 inhibits the movement of tumor cells. (TIFF) [file pone.0051797.s008.tiff]
